# Therapeutic Potential of Sodium Selenite Application for Promoting Radioactive Iodine Avidity in Papillary Thyroid Cancer

**DOI:** 10.1155/bca/3598919

**Published:** 2026-01-27

**Authors:** Ji Min Oh, Ramya Lakshmi Rajendran, Prakash Gangadaran, Chae Moon Hong, Byeong-Cheol Ahn

**Affiliations:** ^1^ Department of Nuclear Medicine, School of Medicine, Kyungpook National University, Daegu, 41944, Republic of Korea, knu.ac.kr; ^2^ Cardiovascular Research Institute, Kyungpook National University, Daegu, 41944, Republic of Korea, knu.ac.kr; ^3^ BK21 FOUR KNU Convergence Educational Program of Biomedical Sciences for Creative Future Talents, Department of Biomedical Science, School of Medicine, Kyungpook National University, Daegu, 41944, Republic of Korea, knu.ac.kr; ^4^ Department of Nuclear Medicine, Kyungpook National University Hospital, Daegu, 41404, Republic of Korea, knu.ac.kr

**Keywords:** papillary thyroid cancer, radioactive iodine avidity, radioactive iodine therapy, sodium iodide symporter, sodium selenite

## Abstract

**Objective:**

Radioactive iodine therapy is a mainstay for recurrent and metastatic differentiated thyroid cancer. However, a substantial portion of differentiated thyroid cancer patients exhibits dedifferentiation status with a lack of sodium iodide symporter functionality and expression, as well as downregulated thyroid‐specific proteins and transcription factors. Eventually, this status is connected to the failure of radioactive iodine therapy with an overall poor prognosis. Selenium, an essential trace element, has antitumor, antioxidant, immunomodulatory, and antiviral activities and is required for thyroid hormone synthesis and metabolism, and it was reported that sodium selenite induces radioactive iodine uptake in thyroid tissue in rats. However, the relationship between sodium selenite and differentiation markers in differentiated thyroid cancer remains unclear.

**Methods:**

We investigated whether sodium selenite enhances radioactive iodine avidity and reinforces ^131^I therapeutic effects in papillary thyroid cancer cells. We also analyzed changes in selected signaling pathways and factors induced by sodium selenite treatment.

**Results:**

Sodium iodide symporter, thyroid‐specific proteins, and transcription factors were upregulated by sodium selenite, increasing radioactive iodine avidity and radioactive iodine‐mediated cytotoxicity in papillary thyroid cancer cells. Sodium selenite downregulated the MAPK, PI3K–AKT, and GSK‐3β/β‐catenin signaling pathways.

**Conclusion:**

Sodium selenite may serve as a promising adjunct to enhance radioactive iodine avidity in papillary thyroid cancer cells.

## 1. Introduction

According to the statistical analysis of all cancer types from GLOBOCAN 2020 produced by the International Agency for Research on Cancer (IARC), the incidence of thyroid cancer is ranked high among 33 types of cancer [1]. Differentiated thyroid cancer (DTC), including papillary thyroid cancer (PTC), accounts for > 90% of cases and generally has an excellent prognosis with standard management, including thyroidectomy, radioactive iodine (RAI) therapy, and thyroid‐stimulating hormone (TSH) suppression [2]. Nonetheless, locoregional recurrence and distant metastasis unexpectedly occur during the initial work‐up or follow‐up. A retrospective cohort study reported that distant metastasis is a crucial determinant of overall and disease‐specific survival in DTC [3]. DTC patients with unresectable metastases can be treated, and occasionally cured, with RAI; however, approximately two‐thirds eventually do not respond to RAI because of dedifferentiation, with 10‐year survival of about 10% [4].

RAI avidity can be increased by upregulating iodide‐metabolizing proteins. Enhancing RAI avidity through redifferentiation may enable salvage management of RAI‐refractory DTC. Many studies have attempted to improve RAI‐mediated therapeutic effects via pharmacologic interventions in thyroid cancer, but most have shown limited effectiveness or no clinical benefit [5]. Although TSH stimulation can increase RAI avidity in thyroid cancer [6], some patients with DTC metastases still exhibit no RAI avidity after adequate administration [7]. Therefore, a clinically practical pharmacologic intervention that can be routinely applied to enhance RAI avidity in DTC is currently lacking. Because increased RAI avidity improves the therapeutic effect of RAI, strategies to increase avidity in DTC are urgently needed.

Selenium is an indispensable trace element whose biological functions are mediated by selenoproteins [8]. Several studies suggested the significance of selenium as a cancer preventive agent because the intake of low‐dose selenium has negative relevance to the risk of cancers [9, 10]. In addition, selenium administration has produced anticancer effects, including upregulation of tumor‐suppressor proteins, alterations in DNA methylation, cell cycle arrest, induction of apoptosis, and inhibition of angiogenesis [10].

Selenium is essential for thyroid hormone synthesis and metabolism, and the thyroid contains the highest selenium concentration per unit tissue among human organs [8, 11]. Accordingly, the relationship between selenium status and thyroid cancer has been extensively investigated. Several studies have reported lower serum selenium concentrations in patients with thyroid cancer than in healthy controls [12–14]. In PTC, serum selenium levels were inversely associated with the number of lymph node metastases [15]. Chadha et al. reported that sodium selenite—commonly used as a selenium source—enhanced RAI uptake and TSH levels and prolonged the biological half‐life of RAI in both euthyroid and hypothyroid animals [16]. However, whether selenium modulates thyroid cancer biology—specifically RAI avidity and RAI‐mediated cytotoxicity—has not been examined.

In the present study, we evaluated whether sodium selenite increases RAI avidity in PTC cells and investigated the underlying molecular mechanisms.

## 2. Materials and Methods

### 2.1. Cell Culture

BHP10‐3SCp, a tumorigenic subclone of BHP10‐3 PTC cells harboring the RET/PTC rearrangement, was kindly provided by Dr. Soon‐Hyun Ahn (Seoul National University College of Medicine, Seoul, Republic of Korea) and Dr. Gary L. Clayman (MD Anderson Cancer Center, Houston, TX) [17]. Cells were maintained in a RPMI 1640 medium (HyClone, Logan, UT, USA) supplemented with 10% fetal bovine serum (FBS; Gibco, Grand Island, NY, USA) and 1% penicillin–streptomycin (HyClone) in a humidified incubator at 37°C with 5% CO_2_.

### 2.2. Reagents

Sodium selenite was purchased from Sigma (St. Louis, MO, USA). Sodium selenite was dissolved in double‐distilled water (ddH_2_O) to prepare a 1‐mM stock solution and stored at −20°C.

### 2.3. siRNA Transfection

Scrambled siRNA and NIS siRNA were purchased from Dharmacon (Lafayette, CO, USA). The siRNAs were dissolved in siRNA buffer (Dharmacon) and prepared as 250 μM stocks. BHP10‐3SCp cells (7 × 10^4^ cells/well) were seeded in 24‐well plates and incubated at 37°C with 5% CO_2_. The next day, the medium was replaced with Opti‐MEM (Gibco, Waltham, MA, USA) and equilibrated for 1 h. Subsequently, 40 nM siRNA was delivered with DharmaFECT transfection reagent (Dharmacon) and cells were incubated for 48 h, as previously described [18].

### 2.4. Cell Viability Assay

Cells (5 × 10^3^ cells/well) were seeded in 96‐well plates in a medium containing 2.5% FBS and 1% penicillin–streptomycin and incubated overnight at 37°C with 5% CO_2_. The next day, cells were treated with sodium selenite (0–20 μM) and incubated for 24, 48, and 72 h. Cell Counting Kit‐8 (CCK‐8) reagent (Dojindo Molecular Technologies, Inc., Rockville, MD, USA) was added and incubated for 2 h. Absorbance at 450 nm was measured using a microplate reader (Multiskan SkyHigh; Thermo Fisher Scientific, Rockford, IL, USA). Cell viability was calculated relative to an average of the control and represented as a percentage (%). The half‐maximal inhibitory concentration (IC_50_) was calculated with GraphPad Prism, version 10.2.3 (GraphPad Software, Boston, MA, USA).

### 2.5. Bioluminescence Imaging

The pNIS‐Fluc2‐TurboFP635‐pCMV‐Rluc plasmid was transfected into BHP10‐3SCp cells, as previously described [19]. After establishing stable cell lines, cells were exposed to 0–2.5 μM sodium selenite for 72 h. After 72 h, 10 μg/mL h‐coelenterazine was added to quantify CMV promoter activity via Renilla luciferase (Rluc) signal using an IVIS Lumina III instrument (PerkinElmer, Wellesley, MA, USA). NIS promoter activity was quantified from the firefly luciferase 2 (Fluc2) signal after addition of 150 μg/mL D‐luciferin using the same instrument.

### 2.6. RNA Extraction

BHP10‐3SCp cells were exposed to 1.25 μM sodium selenite for 72 h. Total RNA was extracted using TRIzol reagent (Invitrogen, San Diego, CA, USA) according to the manufacturer’s instructions. RNA concentration was measured with a NanoDrop spectrophotometer (Thermo Fisher Scientific).

### 2.7. Real‐Time Quantitative Reverse Transcription Polymerase Chain Reaction (qRT‐PCR) Analysis

The extracted RNA was converted to cDNA using a High Capacity cDNA Reverse Transcription Kit (Applied Biosystems, Foster City, CA, USA) according to the manufacturer’s protocol. qRT‐PCR analysis was performed on a CFX96 Touch Real‐Time PCR detection system (Bio‐Rad Laboratories, Inc., Hercules, CA, USA) using SsoAdvancedTM Universal SYBR Green Supermix (Bio‐Rad Laboratories, Inc.) according to the manufacturer’s instructions. Primers for *NIS*, *thyroperoxidase* (*TPO*), *TSH receptor* (*TSHR*), *thyroid transcription factor-1* (*TTF-1*), and *paired box-8* (*PAX-8*) were used to *assess* the effect of sodium selenite on mRNA expression. *β-Actin* served as the internal control for relative qRT‐PCR. The 2^−ΔΔCt^ method was used to calculate relative expression levels of target genes. Primer sequences are listed in Supporting Table S1.

### 2.8. Protein Extraction

Protein extraction from whole‐cell lysates was performed as previously described [18]. For subsequent experiments, cells were treated with 1.25 μM sodium selenite for 72 h. Cells were washed with ice‐cold phosphate‐buffered saline (PBS). After PBS removal, cells were detached with trypsin–EDTA (0.25% trypsin with 2.25 mM EDTA) for 3 min at 37°C with 5% CO_2_ and then quenched with complete culture medium. Cells were centrifuged at 1500 × g for 3 min, and the supernatant was discarded. Pellets were lysed in radioimmunoprecipitation assay (RIPA) buffer (Thermo Fisher Scientific) containing protease and phosphatase inhibitors (Thermo Fisher Scientific). Lysates were briefly vortexed three times at 10‐min intervals and subsequently centrifuged at 13,000 × g for 20 min at 4°C. Supernatants were transferred to fresh tubes.

Both plasma membrane and cytoplasmic proteins were extracted using the Minute Plasma Membrane Protein Isolation and Cell Fractionation Kit (Invent Biotechnologies, Plymouth, MN, USA). Cell pellets were suspended in Buffer A and transferred to a filter cartridge. After high‐speed centrifugation for 30 s, intact nuclear fractions were collected. The supernatant was then centrifuged at high speed for 30 min; the resulting supernatant was retained as the cytoplasmic fraction. Pellets were resuspended in Buffer B and centrifuged at 7800 × g for 5 min. The supernatant was transferred to new tubes, washed twice with cold PBS, and centrifuged at high speed for 30 min. The pellets, representing the plasma membrane fraction, were dissolved in RIPA buffer containing protease and phosphatase inhibitors. Protein concentrations were determined using the bicinchoninic acid (BCA) assay (Thermo Fisher Scientific).

### 2.9. Western Blots

Western blots were performed as previously described [18]. Briefly, 10% SDS–polyacrylamide gels were prepared. Equal amounts of protein (μg) were mixed with 4× Laemmli sample buffer (Bio‐Rad Laboratories) supplemented with 2‐mercaptoethanol (final 355 mM) and boiled at 95°C for 5 min. Proteins were resolved by SDS–PAGE and transferred to polyvinylidene fluoride (PVDF) membranes (Millipore, Burlington, MA, USA). Membranes were blocked in 3% bovine serum albumin (BSA; genDEPOT, Katy, TX, USA) in Tris‐buffered saline with 0.05% Tween‐20 (TBS‐T; BIOSESANG, Seongnam‐si, Gyeonggi‐do, Korea) for 2 h, then incubated overnight at 4°C with primary antibodies diluted in 0.5% BSA. After washing three times with TBS‐T, membranes were incubated for 1 h at room temperature with horseradish peroxidase (HRP)‐conjugated secondary antibodies (anti‐mouse IgG and anti‐rabbit IgG; Cell Signaling) diluted in 0.5% BSA, followed by three additional TBS‐T washes. Chemiluminescent signals were developed with Amersham ECL Select Western Blotting Detection Reagent (Cytiva, Marlborough, MA, USA) and imaged using a Fusion FX chemiluminescence analyzer (Vilber Lourmat, Marne‐la‐Vallée, France) according to the manufacturer’s instructions. Band intensities were quantified on the same system. Blot images were cropped and prepared in PowerPoint (Microsoft, Redmond, WA, USA); contrast was adjusted for visualization when necessary. Supporting Table 2 lists the primary antibodies.

### 2.10. Antibody Arrays

Phospho‐kinase and cell stress array kits (R&D Systems, Minneapolis, MN, USA) were used according to the manufacturer’s instructions to assess relative phosphorylation and cell stress factor levels in BHP10‐3SCp cells after sodium selenite treatment. Cells were lysed in the provided buffer to prepare 300 μg of protein per set. Membranes were blocked in Array Buffer 1 for 1 h, 2 mL of protein lysate was added to each membrane, and incubation proceeded overnight at 4°C. The next day, membranes were washed three times with a wash buffer, incubated with the reconstituted detection antibody cocktail for 2 h at room temperature, washed three times, and then incubated with streptavidin–HRP for 30 min. After three final washes, the chemi reagent mixture was applied, and spots were detected using the Fusion FX chemiluminescence analyzer.

### 2.11. Immunofluorescence Imaging

BHP10‐3SCp cells (1.5 × 10^5^ cells per well) were seeded in a medium containing 2.5% FBS and 1% penicillin–streptomycin on four‐well chamber slides and incubated for 24 h at 37°C in a humidified atmosphere with 5% CO_2_. The next day, cells were treated with sodium selenite for 72 h. After incubation, cells were washed with PBS for 10 min and fixed with 4% paraformaldehyde (BIOSESANG) for 10 min at room temperature. Cells were rinsed three times with PBS, permeabilized with 0.2% Tween‐20 in PBS for 10 min, and washed three additional times with PBS for 10 min each. Cells were blocked with 3% BSA in PBS for 1 h and incubated overnight at 4°C with anti‐NIS primary antibody (1:100; Abcam, Cambridge, United Kingdom). Cells were then rinsed three times with PBS and incubated with Alexa Fluor 647‐conjugated goat anti‐mouse secondary antibody (1:200; Thermo Fisher Scientific) for 1 h, followed by three PBS washes. Nuclear staining and plasma membrane staining were performed with Hoechst 33342 (1:2000; Thermo Fisher Scientific) and CellBrite Orange with DiI (1:2000; Biotium, Fremont, CA, USA) for 10 min each. After three PBS washes, coverslips were mounted on slides with a VECTASHIELD antifade mounting medium (Vector Laboratories, Burlingame, CA, USA), and images were acquired using a confocal laser microscope (LSM 5exciter; Zeiss, Oberkochen, Germany). Images were adjusted in ZEN 2.3 software (Zeiss) to improve visualization by modifying channel colors and scale bars.

### 2.12. 125I Uptake Assay

The 125I uptake assay was performed as previously described [2]. BHP10‐3SCp cells (7 × 10^4^ cells in 500 μL) were seeded in a medium containing 2.5% FBS and 1% penicillin–streptomycin in 24‐well plates. The next day, cells were treated with 1.25 μM sodium selenite and incubated for 72 h at 37°C in a humidified incubator with 5% CO_2_. After 72 h, cells were washed with prewarmed Hank’s balanced salt solution (HBSS; HyClone) containing 0.5% BSA (bHBSS). Carrier‐free 125I (37 kBq; PerkinElmer Life Sciences, Waltham, MA, USA) and sodium iodide (NaI; 100 μM; specific activity 740 MBq/mM; Sigma) were then added, and cells were incubated for 30 min at 37°C with 5% CO_2_. For inhibition studies, cells were pretreated with potassium perchlorate (KClO_4_; 50 μM; Sigma) for 30 min before addition of 125I to block uptake. 125I uptake values are expressed as counts per minute (cpm) per μg protein.

### 2.13. 131I Clonogenic Assay

The 131I clonogenic assay was performed to assess the redifferentiation effect of sodium selenite in four groups: control, 131I alone, sodium selenite alone, and 131I after sodium selenite pretreatment. BHP10‐3SCp cells (2 × 10^5^ in 2 mL) were seeded in six‐well plates in a medium containing 2.5% FBS and 1% penicillin–streptomycin and incubated for 24 h at 37°C in 5% CO_2_. Cells then received sodium selenite for 72 h. Scrambled or NIS siRNA‐transfected cells were included to assess NIS dependence. After incubation, the medium was removed and cells were rinsed twice with bHBSS. Cells were then incubated for 7 h at 37°C with or without 50 μCi/mL 131I (KIRAMS, Seoul, Korea) supplemented with 30 μM NaI. Cells were washed twice with bHBSS, trypsinized, counted, and reseeded in six‐well plates at 1 × 10^3^ cells in 2 mL per well. Plates were incubated at 37°C in 5% CO_2_ until colonies formed. Colonies were washed twice with PBS, fixed in acetic acid–methanol (1:7), stained with 0.05% crystal violet for 1 h, and rinsed with tap water. Colonies with more than 50 cells were counted. Survival fraction (%) was calculated as the plating efficiency (PE) of the treated sample divided by the PE of the untreated control.

### 2.14. Statistical Analysis

Data are presented as mean ± standard deviation. Between‐group differences were assessed using Student′s *t*‐test. Analyses were performed in the GraphPad Prism, version 10.2.3. Values of *p* < 0.05 were considered statistically significant.

## 3. Results

### 3.1. Cell Viability Effect of Sodium Selenite Treatment on PTC Cells

Before assessing changes in iodide‐metabolizing protein expression after sodium selenite treatment, we optimized the sodium selenite concentration. BHP10‐3SCp cells were treated with graded concentrations for different durations. At 24 and 48 h, cell viability decreased gradually, reaching approximately 70% at 20 μM, the highest concentration tested (24 h: 72.50% ± 4.63%; 48 h: 68.36% ± 4.12%; Figure 1(a)). At 72 h, viability declined markedly beginning at 2.5 μM (2.5 μM: 58.81% ± 6.68%; 5 μM: 57.81% ± 6.02%; 10 μM: 51.47% ± 5.32%; 20 μM: 33.39% ± 4.69%; Figure 1(a)). The IC_50_ at 72 h was 2.706 μM (*R* ^2^ = 0.9591). We therefore selected 0–2.5 μM for 24–72‐h incubations in subsequent experiments.

Figure 1Cell viability and dual reporter gene activity after sodium selenite treatment in thyroid cancer cells. (a) Cell viability assay using CCK‐8. (b) Half‐maximal inhibitory concentration (IC50) of sodium selenite. (c) Schematic of the dual reporter gene system. (d) Bioluminescence imaging (BLI) monitoring of NIS and CMV activities. (e) Quantitative analysis of regions of interest (ROIs) by BLI. Results are mean ± SD. ^∗∗∗^ *p* < 0.001, ^∗∗^ *p* < 0.01, ^∗^ *p* < 0.05 (Student’s *t*‐test). BLI, bioluminescence imaging; SD, standard deviation.

**Figure:**
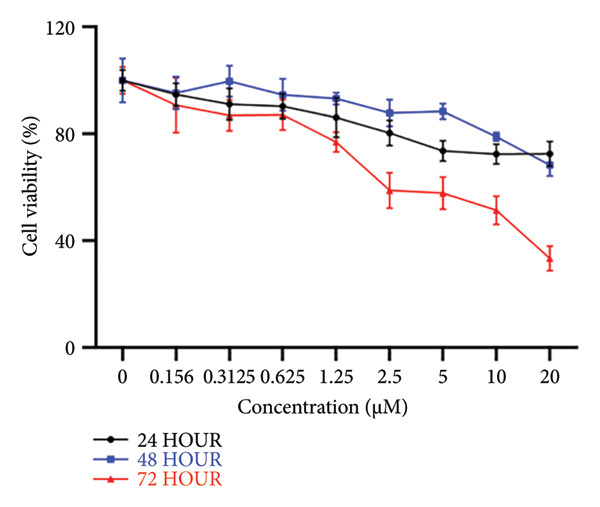
(a)

**Figure:**
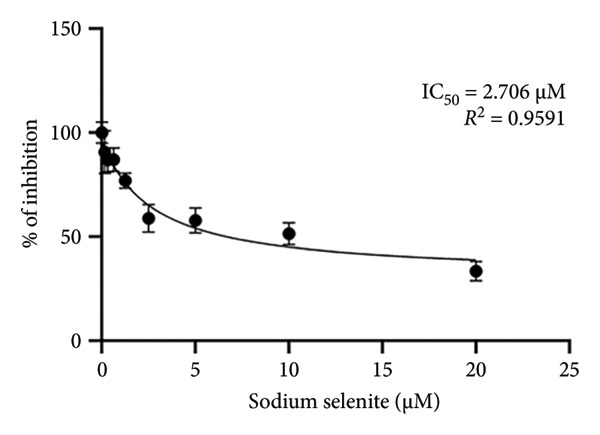
(b)

**Figure:**
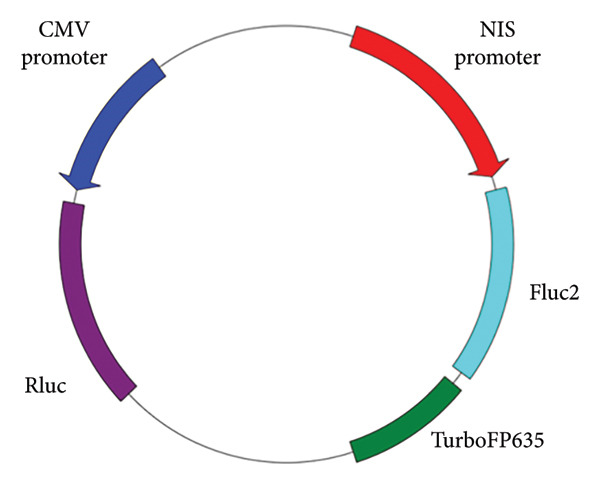
(c)

**Figure:**
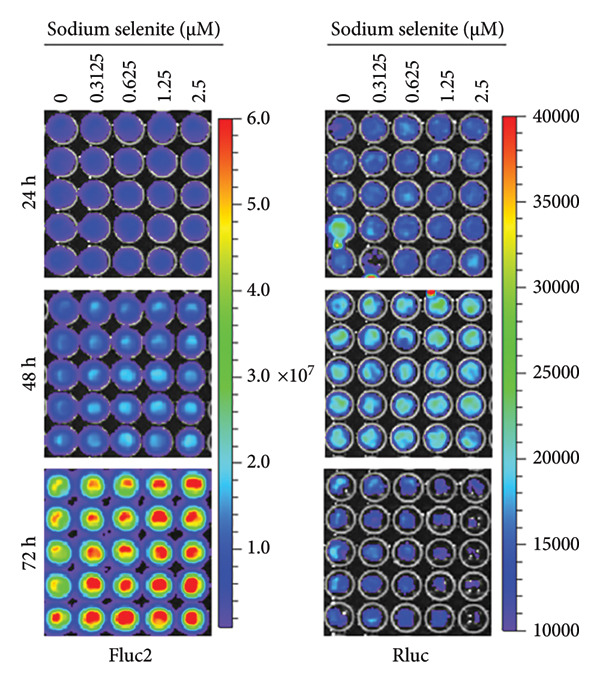
(d)

**Figure:**
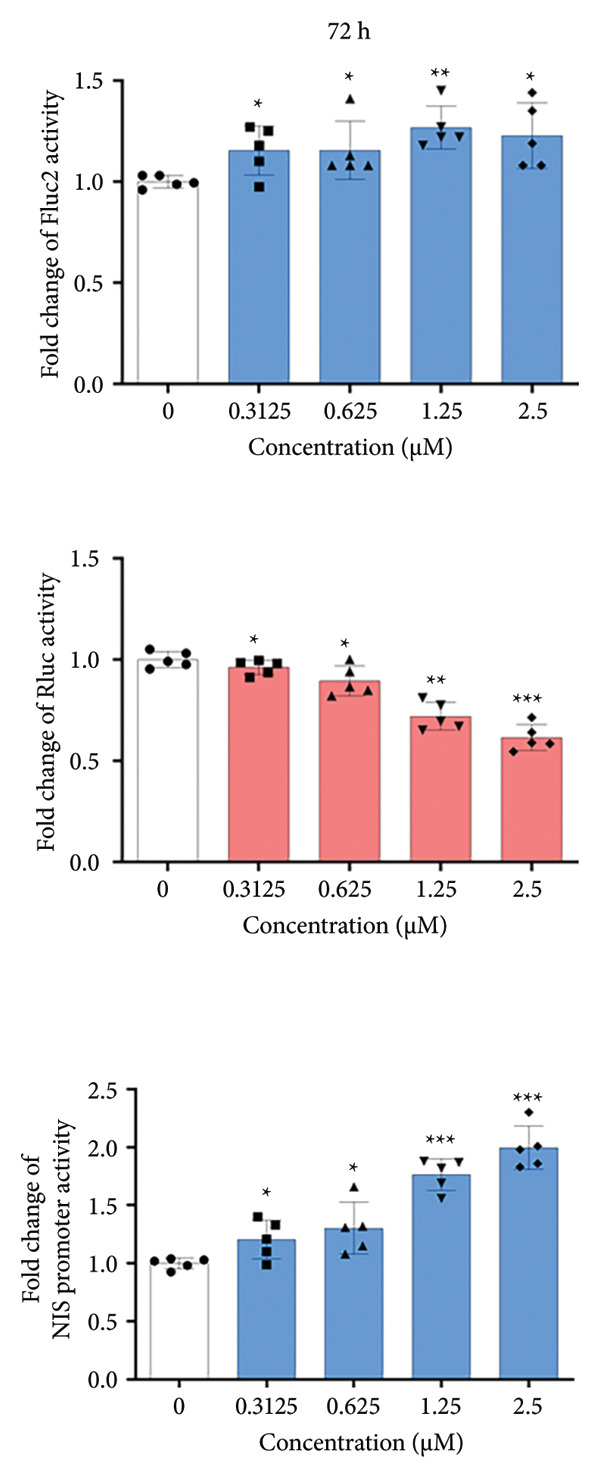
(e)

### 3.2. Monitoring NIS Promoter Activity Following Sodium Selenite Treatment in PTC Cells With the Dual Reporter Gene System

We used a dual reporter gene system to monitor NIS and CMV promoter activities (Figure 1(b)), as described previously [19]. As shown in Figures 1(c) and 1(d) and Supporting Figure 1, Fluc2 signals driven by the NIS promoter were unchanged at 24 h but increased at 48 and 72 h. Rluc activity, which reflects cell viability, did not change at 24 and 48 h but decreased slightly at 72 h. NIS promoter activity was calculated as Fluc2 normalized to Rluc. The highest activity occurred at 72 h (1.25 μM: 1.27 ± 0.10; 2.5 μM: 1.23 ± 0.16; Figure 1(e), upper panel). Accordingly, we used 1.25 μM sodium selenite for 72 h in subsequent experiments.

### 3.3. Robust Augmentation of Genes Regulating Iodide‐Metabolizing Machinery by Sodium Selenite in PTC Cells

NIS, located on the basolateral membrane, mediates iodide uptake in PTC cells. Refractoriness to RAI, a cause of treatment failure in aggressive thyroid cancer, is linked to decreased NIS expression and mislocalization away from the plasma membrane. Other thyroid‐specific genes (*TPO* and *TSHR*) and transcription factors (*PAX-8* and *TTF-1*) are also critical for restoring RAI avidity by coordinating iodide‐metabolizing machinery. We therefore examined whether sodium selenite upregulates these genes. *NIS* expression increased 2.21‐fold relative to the control (Figure 2(a)). *TSHR* and *TPO* rose 1.58‐fold and 1.60‐fold, respectively (Figure 2(b)). Thyroid transcription factors were also elevated (Figure 2(c); *PAX-8*: 1.71 ± 0.097; *TTF-1*: 2.99 ± 1.04). These data indicate that sodium selenite enhances expression of genes governing iodide metabolism in PTC cells.

Figure 2Evaluation of factors regulating iodide‐metabolizing machinery in thyroid cancer cells after sodium selenite treatment. (a) Real‐time quantitative reverse transcription polymerase chain reaction (qRT‐PCR) analysis of *NIS* gene expression. *β-actin* was used as the housekeeping gene. Data are mean ± SD. ^∗∗^ *p* < 0.01 (Student’s *t*‐test). (b) Expression levels of thyroid‐specific genes (*TSHR*, *TPO*) by qRT‐PCR analysis. *β-actin* was used as the housekeeping gene. Data are mean ± SD. ^∗∗^ *p* < 0.01 (Student’s *t*‐test). (c) Changes in thyroid transcription factors (PAX*-*8, TTF*-*1) by qRT‐PCR analysis. *β-actin* was used as the housekeeping gene. Data are mean ± SD. ^∗∗∗^ *p* < 0.001 (Student’s *t*‐test). (d) Western blot analysis showing endogenous NIS expression in whole‐cell lysates with quantitative analysis. GAPDH was used as an internal control. Data are mean ± SD. ^∗∗^ *p* < 0.01 (Student’s *t*‐test). (e) Western blot and quantitative analyses of endogenous NIS expression in cytoplasmic and plasma membrane fractions. GAPDH and caveolin‐1 were used as internal controls. Data are mean ± SD. ^∗^ *p* < 0.05; NS, not significant (Student’s *t*‐test). (f) Immunofluorescence showing NIS localization. Scale bar, 20 μm. (g) Expression of thyroid‐specific proteins with quantitative analysis. GAPDH was used as an internal control. Data are mean ± SD. ^∗^ *p* < 0.05 (Student’s *t*‐test). (h) Expression of thyroid transcription factors with quantitative analysis. GAPDH was used as an internal control. Data are mean ± SD. ^∗∗∗^ *p* < 0.001; ^∗^ *p* < 0.05 (Student’s *t*‐test). SD, standard deviation. Raw data are presented in Supporting Figure 2.

**Figure:**
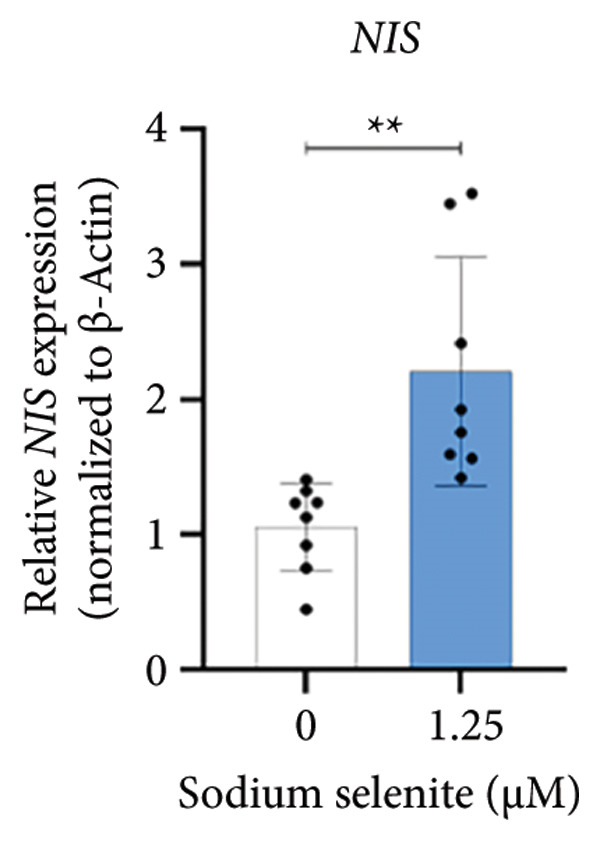
(a)

**Figure:**
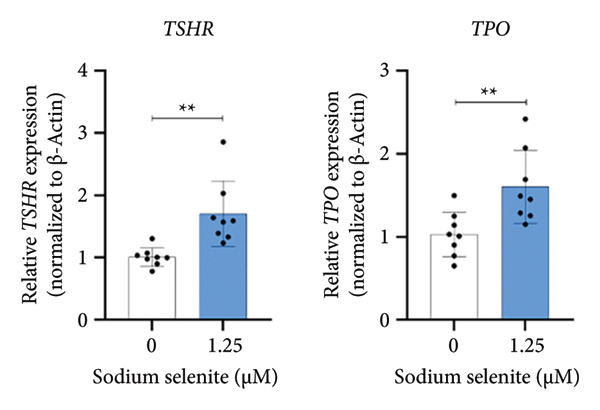
(b)

**Figure:**
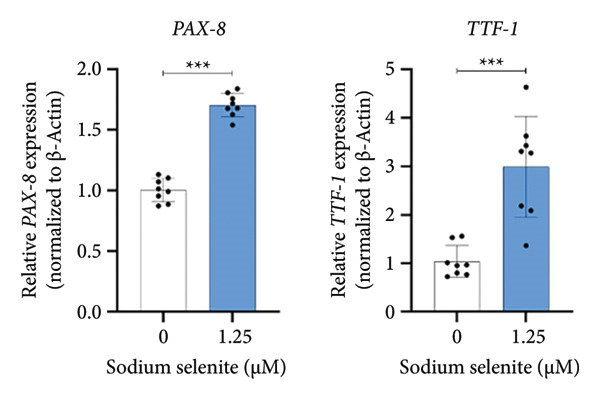
(c)

**Figure:**
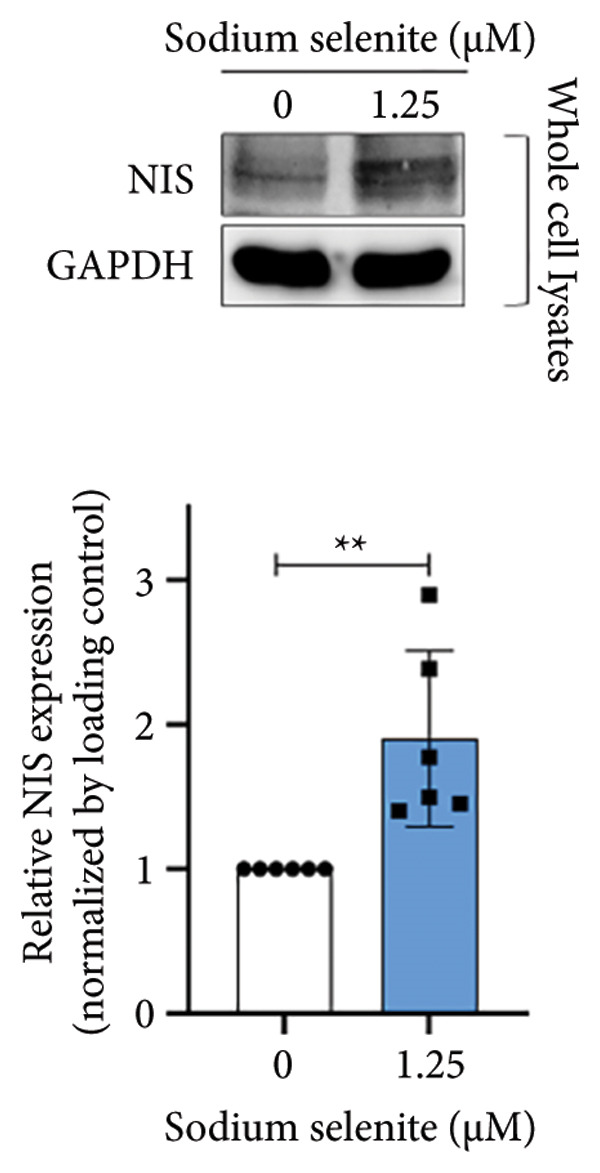
(d)

**Figure:**
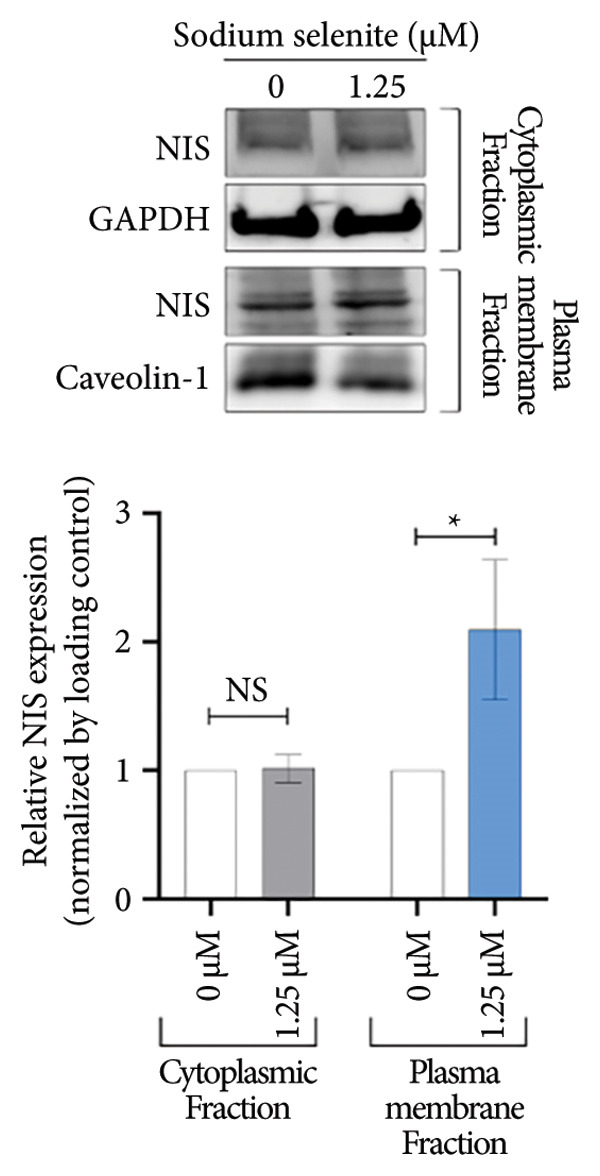
(e)

**Figure:**
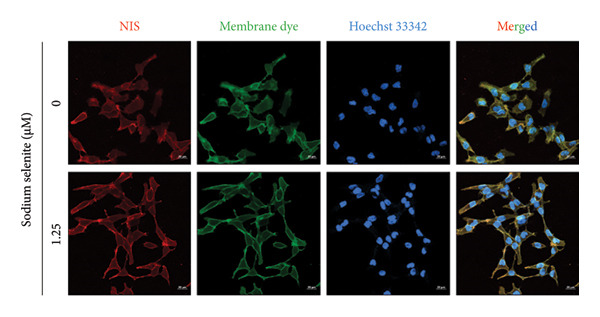
(f)

**Figure:**
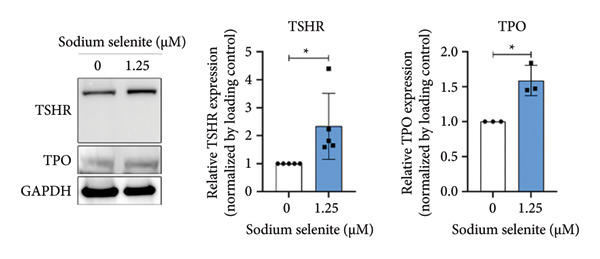
(g)

**Figure:**
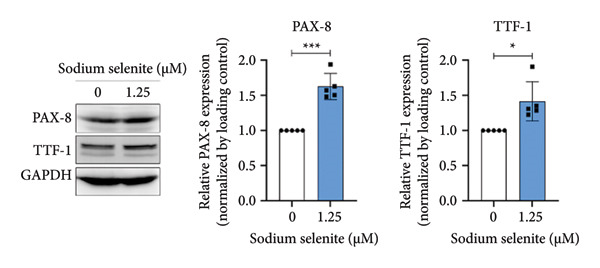
(h)

### 3.4. Identification of Enhanced NIS Expression and Its Plasma Membrane Localization by Sodium Selenite in PTC Cells

We next assessed NIS protein expression. As shown in Figure 2(d), the fully glycosylated, mature NIS band increased after sodium selenite treatment in BHP10‐3SCp cells. Quantification showed a 1.90‐fold increase versus the control. In contrast, lower molecular weight, nonglycosylated NIS forms (< 90 kDa) decreased (Supporting Figure 2). We then evaluated the change of NIS localization between the plasma membrane and cytoplasm using fractionation. Cytoplasmic NIS levels were unchanged (1.02 ± 0.11; Figure 2(e); Supporting Figure 2), whereas the plasma membrane NIS increased (2.09 ± 0.54; Figure 2(e); Supporting Figure 2). Consistent with these findings, immunostaining demonstrated marked enrichment of endogenous NIS at the plasma membrane after sodium selenite treatment relative to the control (Figure 2(f)). Thus, sodium selenite upregulated NIS and promoted its plasma membrane localization.

### 3.5. Influence of Sodium Selenite on Thyroid‐Specific Proteins and Transcription Factors in PTC Cells

In BHP10‐3SCp cells, sodium selenite increased thyroid‐specific proteins relative to the control, including TPO and TSHR (TPO, 1.59 ± 0.22; TSHR, 1.82 ± 0.29; Figure 2(g) and Supporting Figure 2). Sodium selenite also increased the transcription factors PAX‐8 and TTF‐1 (PAX‐8, 1.63 ± 0.19; TTF‐1, 1.41 ± 0.28; Figure 2(h) and Supporting Figure 2). Together, these data indicate that sodium selenite upregulated thyroid‐specific proteins and transcription factors in BHP10‐3SCp cells.

### 3.6. Impact of Sodium Selenite on RAI Avidity and RAI‐Mediated Cytotoxicity in PTC Cells

Guided by these findings, we tested whether sodium selenite enhances RAI avidity and RAI‐mediated cytotoxicity in BHP10‐3SCp cells. Sodium selenite significantly increased RAI avidity (Figure 3(a)), elevating RAI uptake 1.95‐fold versus the control. This increase was abolished by pretreatment with potassium perchlorate (KClO_4_), a competitive NIS inhibitor that blocks iodide transport into thyroid cells. These data indicate that sodium selenite‐induced upregulation of iodide‐handling proteins increases RAI uptake in PTC cells.

Figure 3Effectiveness of sodium selenite in promoting RAI avidity and RAI‐mediated cytotoxicity in thyroid cancer cells. (a) RAI uptake after sodium selenite treatment. KClO_4_ was used as a competitive inhibitor of iodide transport. Data are mean ± SD. ^∗∗^ *p* < 0.01; NS, not significant (Student’s *t*‐test). (b) The 131I clonogenic assay to monitor RAI‐mediated cytotoxicity after sodium selenite pretreatment. (c) Quantitative analysis of the 131I clonogenic assay. Survival fraction (%) was expressed as mean ± SD. ^∗∗∗^ *p* < 0.001, ^∗^ *p* < 0.05 (vs. control); ^###^ *p* < 0.001, ^#^ *p* < 0.05 (vs. sodium selenite); ^$$$^ *p* < 0.001 (vs. 131I) (Student’s *t*‐test).

**Figure:**
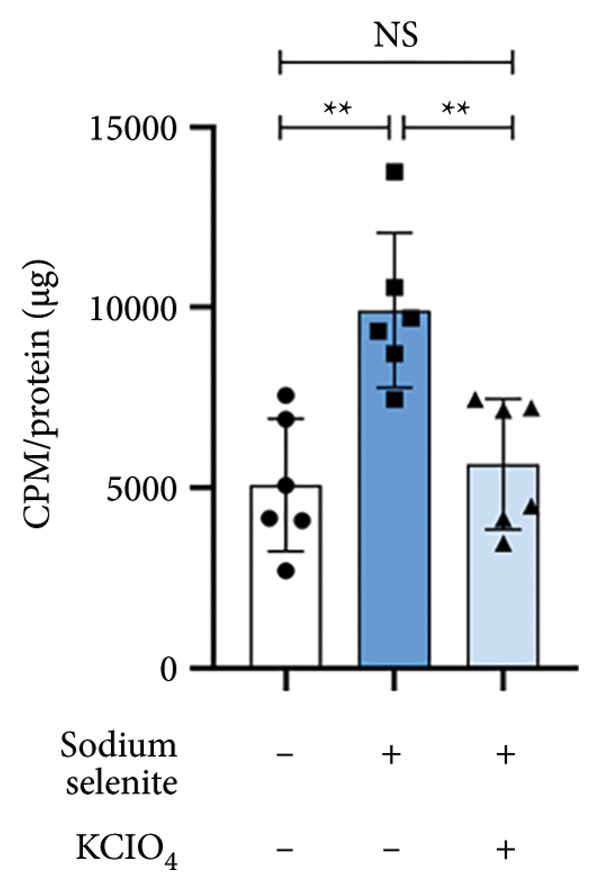
(a)

**Figure:**
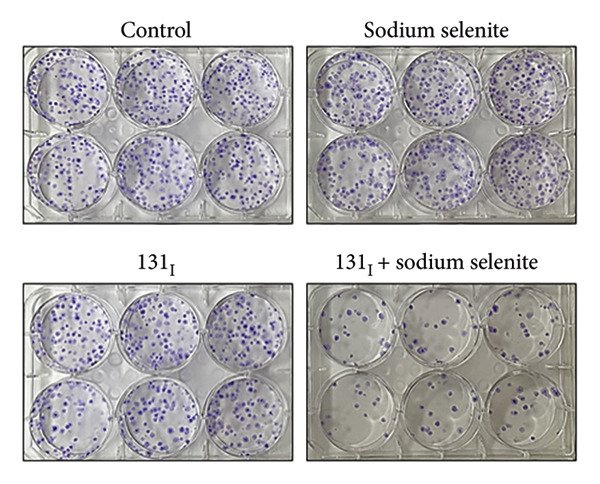
(b)

**Figure:**
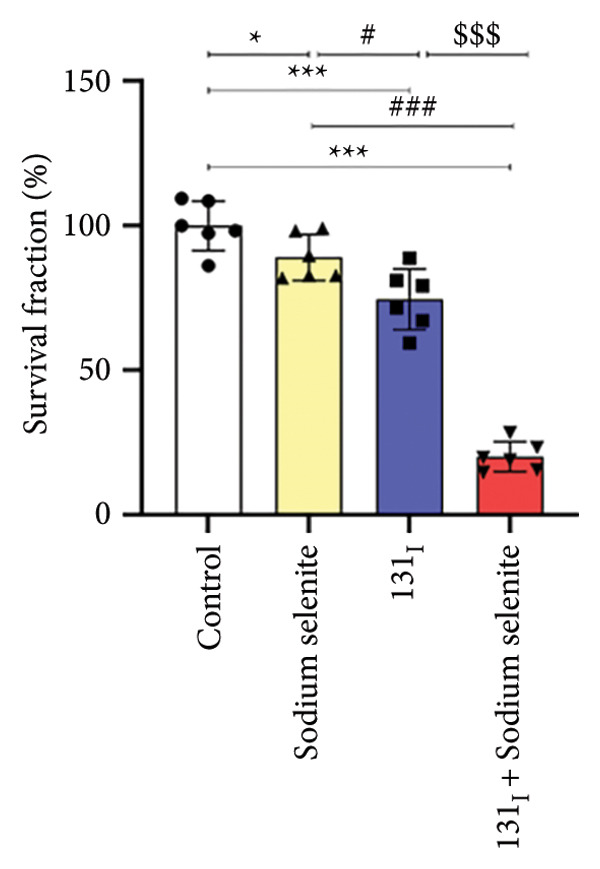
(c)

We next evaluated RAI‐mediated cytotoxicity after sodium selenite pretreatment in PTC cells. As shown in Figure 3(b), sodium selenite alone and 131I alone modestly reduced colony formation relative to the control. By contrast, 131I following sodium selenite pretreatment markedly decreased the colony number. We calculated survival fraction from colony counts in each group. Single‐agent cytotoxicity was < 30% versus the control (sodium selenite, 89.08% ± 7.97%; 131I, 74.57% ± 10.55%; Figure 3(c)). 131I after sodium selenite further decreased survival to 20.11% ± 5.13%. To determine NIS dependence, we knocked down NIS with siRNA. As shown in Supporting Figure 3, 131I after transfection with scrambled siRNA significantly reduced colony number and survival compared with scrambled siRNA alone. NIS knockdown alone and 131I after NIS knockdown did not differ, indicating that 131I efficacy depends on functional NIS. Thus, 131I administered after sodium selenite pretreatment may be a promising strategy to improve therapeutic outcomes in PTC.

### 3.7. Alterations in MAPK and PI3K–AKT Signaling Pathways Induced by Sodium Selenite in PTC Cells

In PTC, activation of MAPK and PI3K–AKT pathways promotes tumorigenesis and dedifferentiation, downregulating iodide‐handling proteins such as endogenous NIS and fostering RAI refractoriness. We therefore examined pathway modulation by sodium selenite that could account for increased iodide‐handling proteins. Sodium selenite reduced ERK and AKT phosphorylation (Figures 4(a), 4(b); Supporting Figure 4). ERK phosphorylation, a downstream MAPK readout, decreased by 55% versus the control (Figure 4(a)). For AKT, phosphorylation at serine 473 (Ser473) and threonine 308 (Thr308) also declined; Ser473 decreased by 66% and Thr308 by 46% (Figure 4(b)). A phospho‐kinase array profiled multiple kinases, including ERK and AKT, and confirmed their inhibition (Figure 4(c)). Sodium selenite also reduced p70 S6 kinase phosphorylation at threonine 389 and threonine 421/serine 424, a downstream mTOR target in the PI3K–AKT pathway (Figure 4(d)). Overall, sodium selenite markedly attenuated MAPK and PI3K–AKT signaling, key pathways in thyroid cancer dedifferentiation.

Figure 4Verification of signaling pathways and cell stress factors after sodium selenite treatment in thyroid cancer cells. (a) Levels of phospho‐ERK (*p*‐ERK) and ERK. β‐actin was used as an internal control. Data are mean ± SD from triplicate experiments. ^∗∗∗^ *p* < 0.001 (Student’s *t*‐test). (b) Expression levels of phospho‐AKT (*p*‐AKT) at Ser473, Thr308 and AKT after sodium selenite treatment. β‐actin was used as an internal control. Data are mean ± SD from triplicate experiments. ^∗∗∗^ *p* < 0.001 (Student’s *t*‐test). (c) Comparison of the phospho‐kinase array between control and sodium selenite treatment. (d) Quantitative analysis of the phospho‐kinase array. (e) Confirmation of the GSK‐3β/β‐catenin signaling pathway after sodium selenite treatment. (f) Quantitative analysis of Western blots. GAPDH, β‐actin, and histone H3 were used as internal controls. Data are mean ± SD from triplicate experiments. ^∗∗^ *p* < 0.01 (Student’s *t*‐test). (g) Comparison of cell stress factors between control and sodium selenite treatment. (h) Quantitative analysis of the cell stress array. Raw data are presented in Supporting Figure 4.

**Figure:**
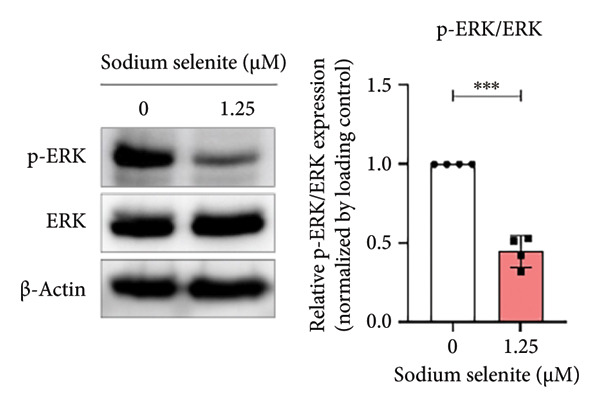
(a)

**Figure:**
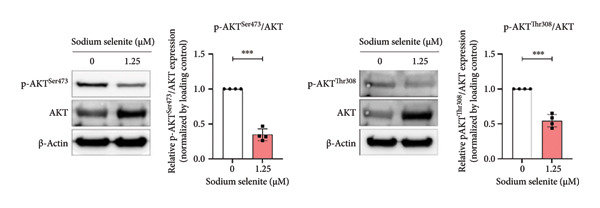
(b)

**Figure:**
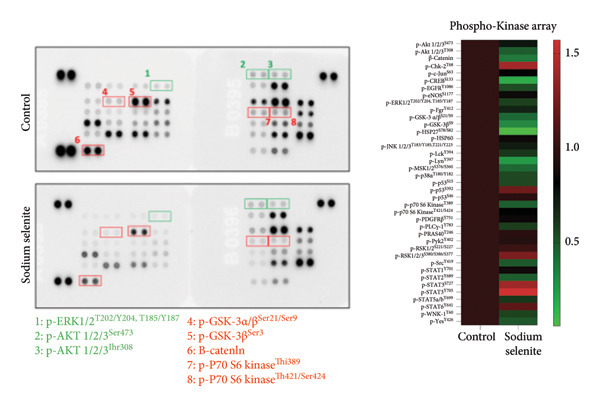
(c)

**Figure:**
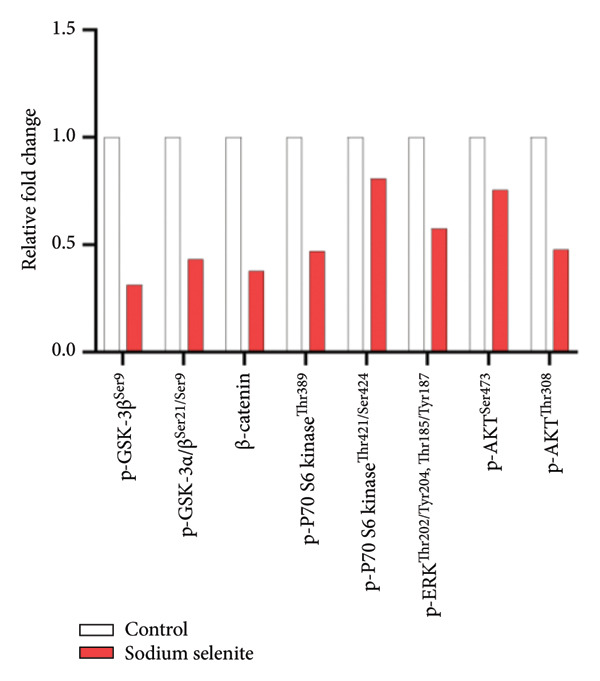
(d)

**Figure:**
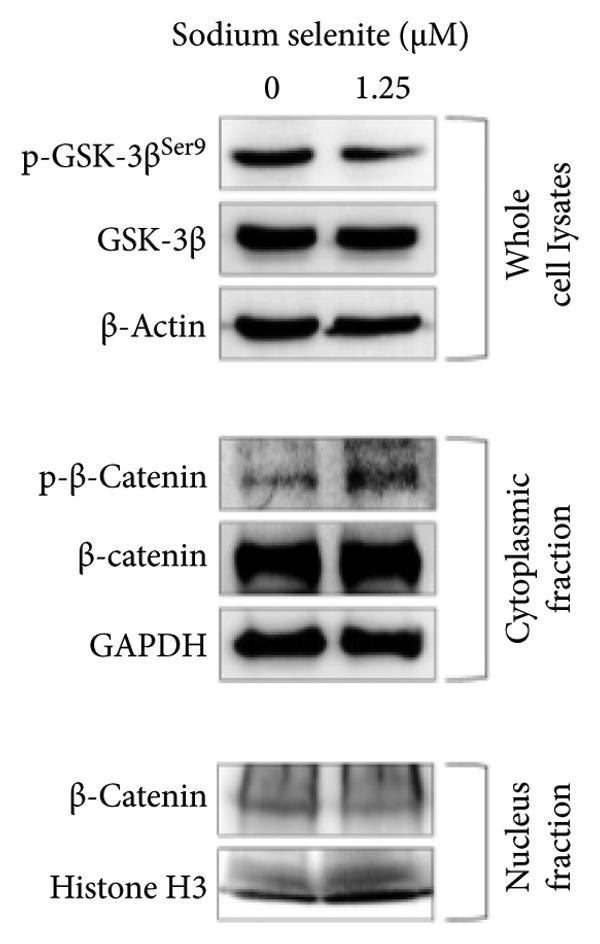
(e)

**Figure:**
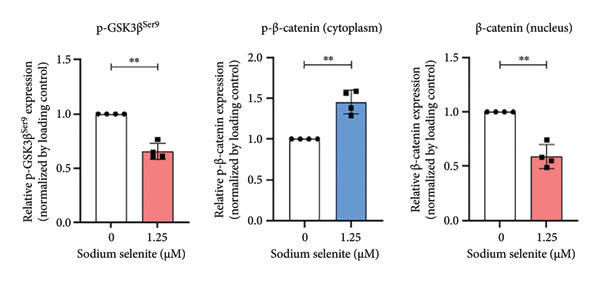
(f)

**Figure:**
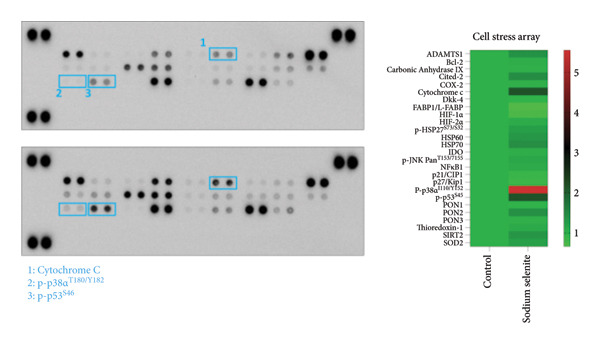
(g)

**Figure:**
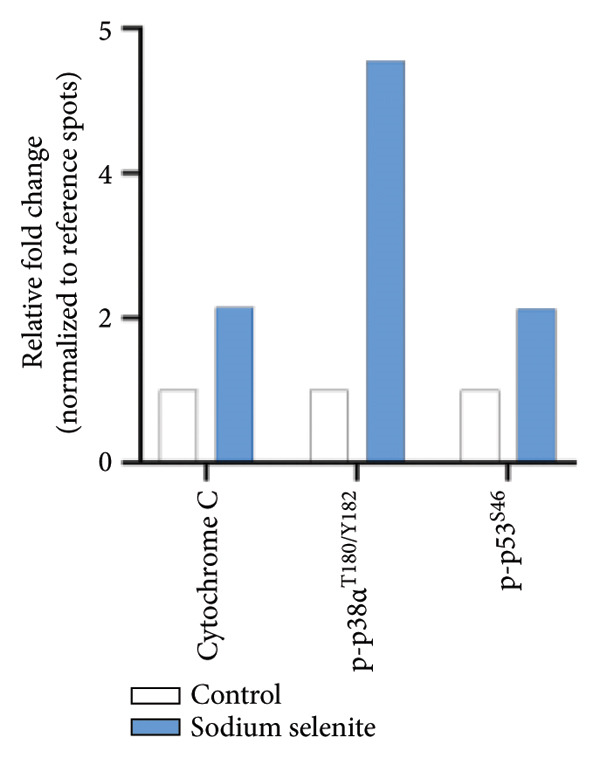
(h)

### 3.8. Influence of Sodium Selenite on the GSK‐3β/β‐Catenin Signaling Pathway in PTC Cells

Sodium selenite affected the GSK‐3β/β‐catenin pathway (Figures 4(c), 4(d) and Supporting Figure 4). Quantitative analysis showed significant decreases in β‐catenin and GSK‐3β phosphorylation after sodium selenite treatment (phosphorylation of GSK‐3β at serine 9, 0.313; phosphorylation of GSK‐3β at serine 21/serine 9, 0.432; β‐catenin, 0.377; Figure 4(d)). We therefore examined proteins in this pathway using whole‐cell lysates. Sodium selenite reduced GSK‐3β phosphorylation at serine 9 versus the control (Figure 4(e); Supporting Figure 4). Sodium selenite increased cytoplasmic phosphorylated β‐catenin, consistent with cytoplasmic β‐catenin degradation, and decreased nuclear β‐catenin. Quantitatively, nuclear β‐catenin and whole‐cell GSK‐3β phosphorylated at serine 9 declined to 0.59 ± 0.11 and 0.66 ± 0.07, respectively, whereas cytoplasmic phosphorylated β‐catenin increased 1.46‐fold (Figure 4(f)). In summary, sodium selenite increased cytoplasmic phosphorylated β‐catenin, indicating enhanced degradation, and reduced GSK‐3β phosphorylation, concomitant with decreased nuclear β‐catenin.

### 3.9. Impact of Sodium Selenite on Cell Stress Factors in PTC Cells

Prior studies have shown that sodium selenite inhibits proliferation by inducing cell cycle arrest and suppresses metastasis through reactive oxygen species (ROS) in multiple cancers [20–23]. Motivated by these findings, we performed a cell stress protein array after sodium selenite treatment in PTC cells (Figure 4(g)). We analyzed the data using a ≥ 2‐fold change threshold between the control and treated groups. As shown in Figure 4(h), sodium selenite increased cytochrome c, p38*α* phosphorylated at threonine 180/tyrosine 182, and p53 phosphorylated at serine 46. Thus, sodium selenite modulated multiple stress response factors in PTC cells.

## 4. Discussion

This study shows that sodium selenite upregulated iodide‐metabolizing proteins and increased 125I uptake and 131I‐mediated cytotoxicity in PTC cells.

Selenium occurs in two main forms: organic (selenomethionine, selenocysteine, and methylselenocysteine) and inorganic (selenite and selenate) [24]. Selenomethionine is actively transported across the intestinal mucosa, whereas selenite is absorbed by passive diffusion. All forms are converted to selenide, which supports selenoprotein synthesis, or to methylated metabolites excreted via the lungs and kidneys [25, 26]. Hydrogen selenide (derived from selenite) and methylselenol (produced from methylselenocysteine) are key antitumor metabolites [24]. Because selenomethionine is nonspecifically incorporated into proteins in place of methionine, it exhibits less antitumor activity than selenite or methylselenocysteine [24]. Sodium selenite has been evaluated as an adjunct to chemotherapy and radiotherapy [27–29]. In a randomized study in non‐Hodgkin lymphoma, adding sodium selenite to standard chemotherapy improved outcomes relative to chemotherapy alone [27]. Song et al. tested sodium selenite plus chemotherapy in a Phase 1 trial in gynecologic cancers [28]. In a Phase 1 study combining sodium selenite with palliative radiotherapy for metastatic cancers, treatment enhanced radiotherapy effects, stabilized disease, and reduced serum prostate‐specific antigen [29]. Sodium selenite has also been investigated as a supportive agent to mitigate cancer‐related lymphedema and radiotherapy‐induced salivary gland dysfunction. As noted above, sodium selenite reduced radiotherapy side effects in metastatic cancer patients [29]; specifically, it alleviated breast cancer‐related lymphedema [30]. In thyroid cancer patients receiving RAI therapy, sodium selenite supplementation protected salivary gland function and secretion [31, 32]. A systematic review of randomized trials summarized the benefits of selenium for RAI‐induced salivary gland dysfunction in RAI‐treated DTC patients [33].

Sodium selenite increases RAI uptake in thyroid tissue and lowers TSH levels [16]. Multiple reports indicate that sodium selenite exerts diverse effects in benign and malignant thyroid cells—enhancing radiotherapy, increasing RAI uptake, inducing cell cycle arrest and apoptosis, and limiting oxidative stress and cell damage [16, 20, 34, 35]. Leoni et al. further showed that sodium selenite augments TSH‐induced NIS expression by modulating redox status in normal thyrocytes [36]. However, to our knowledge, no data have demonstrated that sodium selenite upregulates iodide‐metabolizing proteins in PTC. We therefore investigated whether sodium selenite could restore RAI avidity in PTC by upregulating these proteins.

In this study, sodium selenite increased NIS promoter activity in thyroid cancer cells. We therefore assessed NIS expression levels and observed an increase relative to the control. Importantly, NIS dysfunction can arise not only from reduced expression but also from impaired trafficking to, or retention at, the plasma membrane [2, 5]. Several studies have reported predominantly cytoplasmic, rather than membranous, NIS staining in thyroid cancer. For example, Dohan et al. found that 70% of thyroid cancer specimens exhibited elevated endogenous NIS that was largely intracellular [37–39]. Thus, efficient targeting of NIS to the plasma membrane is essential for effective RAI therapy, in addition to upregulating NIS expression. In our experiments, sodium selenite markedly increased plasma membrane NIS without altering cytoplasmic NIS.

To maximize RAI avidity and cytotoxicity, other thyroid‐specific proteins (TPO and TSHR) and transcription factors (PAX‐8 and TTF‐1) that govern iodide handling, alongside NIS, must also be highly expressed [5, 40, 41]. Their aberrant silencing is a major driver of RAI refractoriness [5]. Numerous agents targeting MAPK [42, 43], HDAC [44–46], PI3K–AKT [43, 47], Notch [48], PPARγ [49], the retinoic acid receptor [50], and reverse transcriptase [51] have been evaluated to overcome this refractoriness. In PTC cells, sodium selenite significantly increased the expression of multiple thyroid‐specific proteins and transcription factors.

RAI therapy is a mainstay first‐line option for unresectable recurrence and/or metastasis in DTC. However, some patients either fail to respond initially or gradually lose RAI avidity, demonstrating a deficiency because of deficiencies in iodide‐metabolizing proteins. Thus, strategies that upregulate this machinery and restore RAI avidity are essential. In a BRAF‐driven mouse model, small‐molecule MAPK inhibitors increased proteins involved in iodide metabolism and enhanced RAI uptake in thyroid tissue [42]. These preclinical findings motivated clinical studies. Vemurafenib restored RAI uptake in RAI‐refractory thyroid cancer and produced tumor regression after RAI therapy [52]. Selumetinib similarly recovered RAI avidity, enabling RAI therapy that stabilized disease [53]. Clinical use of the PPARγ agonist rosiglitazone increased RAI avidity and improved tumor regression after subsequent RAI therapy [54, 55]. Retinoic acids improved RAI uptake in 19 out of 27 patients and yielded tumor regression or disease stabilization in 7 out of 17 treated patients [56]. Collectively, enhancing or restoring RAI avidity is critical for successful treatment of RAI‐refractory thyroid cancer.

Because sodium selenite upregulated iodide‐metabolizing proteins, we confirmed that it increased RAI avidity in PTC cells relative to controls. Prior studies have reported antitumor effects of sodium selenite in thyroid cancer [20, 34]. Consistent with these reports, our study showed cytotoxic activity in PTC cells. Moreover, sodium selenite pretreatment before RAI exposure produced synergistic, not additive, cytotoxicity. Ultimately, sodium selenite pretreatment enhanced RAI‐mediated cytotoxicity by increasing NIS expression and its plasma membrane localization, likely via upregulation of thyroid‐specific proteins and transcription factors. Thus, in vivo studies of sodium selenite to enhance the therapeutic effect of RAI in thyroid cancer are warranted to facilitate clinical translation.

**Figure 5:**
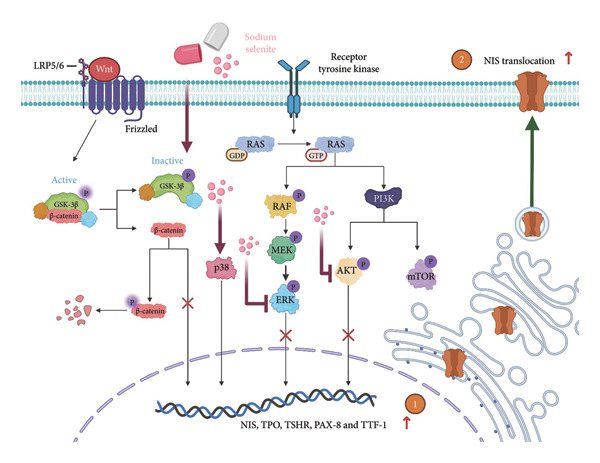
Schematic of increased RAI avidity after sodium selenite treatment in thyroid cancer cells.

Genetic alterations that activate the MAPK and PI3K–AKT pathways drive tumorigenesis, progression, and dedifferentiation in DTC [57]. These alterations also downregulate NIS, thyroid‐specific proteins, and transcription factors, leading to RAI treatment failure [5]. Therefore, we evaluated sodium selenite‐induced changes in key downstream targets of the MAPK and PI3K–AKT pathways implicated in thyroid cancer progression and dedifferentiation. Sodium selenite significantly decreased ERK and AKT phosphorylation (Figure 5).

Prior studies indicate that sodium selenite modulates multiple signaling mechanisms—including ROS–NF‐κB, Hedgehog, endoplasmic reticulum stress, oxidative stress, and GSK‐3β/β‐catenin—in addition to MAPK and PI3K–AKT [21, 58–61]. Accordingly, we used a phospho‐kinase array to compare the control and sodium selenite‐treated cells. Sodium selenite markedly inhibited ERK and AKT phosphorylation, which we confirmed by Western blotting. It also downregulated p70 S6 kinase, a downstream target of mTOR in the PI3K–AKT pathway. Moreover, sodium selenite targeted the GSK‐3β/β‐catenin pathway, decreasing GSK‐3β Ser9 phosphorylation and β‐catenin levels in thyroid cancer cells. GSK‐3β is phosphorylated at Ser9 by AKT, protein kinase A (PKA), or integrin‐linked kinase (ILK), which inactivates the enzyme [62]. Consequently, β‐catenin translocate to the nucleus and activates transcription factors. Garcia‐Rostan et al. suggested that elevated nuclear β‐catenin contributes to tumorigenesis and dedifferentiation in thyroid cancer [63, 64]. We therefore analyzed the GSK‐3β/β‐catenin pathway in whole‐cell lysates with or without sodium selenite. Nuclear β‐catenin was substantially downregulated following reduced GSK‐3β Ser9 phosphorylation, whereas cytoplasmic β‐catenin phosphorylation, indicative of degradation, increased. Thus, sodium selenite targets the MAPK, PI3K–AKT, and GSK‐3β/β‐catenin pathways in PTC.

Cancer cells engage several stress response pathways, including oxidative, metabolic, mechanical, and genotoxic responses. Targeting these pathways has therapeutic potential [65]. Selenium protects against oxidative stress and cellular damage [35, 36] and modulates redox status and cellular stress in nonthyroid cancers [21, 60]. We therefore examined representative stress markers after sodium selenite treatment in thyroid cancer cells. In PTC cells, sodium selenite altered cytochrome c and the phosphorylation of p38*α* and p53. Yan et al. reported that indolequinone induced a thioredoxin redox shift and activated p38 and JNK phosphorylation to promote apoptosis in pancreatic cancer [66]. Moreover, Kogai et al. linked p38 pathway activation to increased NIS expression in breast cancer cells [67]. Although such associations have not been reported in thyroid cancer, studies suggest that targeting p38 phosphorylation can inhibit proliferation and induce p53‐mediated apoptosis [68, 69]. Consistently, our results showed that sodium selenite altered p53 expression and p38 phosphorylation in PTC cells. A review by Cazarin et al. concluded that oxidative stress and redox status regulate NIS expression [70]. Accordingly, sodium selenite influenced stress pathways, ultimately altering NIS expression. Future studies should investigate interactions between stress mediators, including p53 and p38, and proteins that regulate the iodide‐metabolizing machinery.

## 5. Conclusion

In summary, sodium selenite increased the expression of iodide‐metabolizing proteins, enhanced RAI avidity, and augmented RAI‐mediated cytotoxicity in PTC. Sodium selenite pretreatment may therefore be used to enhance the therapeutic efficacy of RAI in PTC.

**Nomenclature**
RAI: Radioactive iodine
DTC: Differentiated thyroid cancer
NIS: Sodium iodide symporter
PTC: Papillary thyroid cancer
TSH: Thyroid‐stimulating hormone
TSHR: TSH receptor
TPO: Thyroperoxidase
ROS: Reactive oxygen species
Rluc: Renilla luciferase
Fluc2: Firefly luciferase 2

## Conflicts of Interest

The authors declare no conflicts of interest.

## Author Contributions

Conceptualization, Ji Min Oh and Byeong‐Cheol Ahn; methodology, Ji Min Oh and Prakash Gangadaran; software, Ji Min Oh, Prakash Gangadaran, Ramya Lakshmi Rajendran, and Chae Moon Hong; validation, Ji Min Oh, Chae Moon Hong, and Byeong‐Cheol Ahn; formal analysis, Ji Min Oh, Prakash Gangadaran, Ramya Lakshmi Rajendran, and Byeong‐Cheol Ahn; investigation, Ji Min Oh, Prakash Gangadaran, Ramya Lakshmi Rajendran, and Chae Moon Hong; resources, Byeong‐Cheol Ahn; data curation, Ji Min Oh and Byeong‐Cheol Ahn; writing–original draft, Ji Min Oh and Byeong‐Cheol Ahn; writing–review and editing, Ji Min Oh, Prakash Gangadaran, Ramya Lakshmi Rajendran, C.M.H., and Byeong‐Cheol Ahn; visualization, Ji Min Oh, Prakash Gangadaran, Ramya Lakshmi Rajendran, Chae Moon Hong, and Byeong‐Cheol Ahn; supervision, Byeong‐Cheol Ahn; funding acquisition, Byeong‐Cheol Ahn.

## Funding

This work was supported by the National Research Foundation of Korea (NRF) grants funded by the Korea government (MSIT; NRF‐2022R1A2C2005057 and NRF‐2022R1C1C2003085) and by the Biomedical Research Institute Grant, Kyungpook National University Hospital (2021).

## Supporting Information

Supporting Table 1: Primer sequences for qRT‐PCR analysis.

Supplementary Table 2: Antibodies used for Western blot analysis.

Supporting Figure 1: Quantitative analysis of bioluminescence imaging after sodium selenite treatment of BHP10‐3SCp cells expressing a dual reporter gene system.

Supporting Figure 2: Uncropped Western blot images for Figure 2.

Supporting Figure 3: 131I clonogenic assay in functional dependency of NIS with scrambled siRNA or NIS siRNA treatment in BHP10‐3SCp cells.

Supporting Figure 4: Uncropped Western blot images for Figure 4.

## Supporting information


**Supporting Information** Additional supporting information can be found online in the Supporting Information section.

## Data Availability

Data and materials are available from the corresponding authors upon reasonable request.
