# Supplementary material for: Therapeutic Potential of Sodium Selenite Application for Promoting Radioactive Iodine Avidity in Papillary Thyroid Cancer
Source: Bioinorg Chem Appl. 2026 Jan 27;2026:3598919. doi: 10.1155/bca/3598919 (PMC12836042; doi:10.1155/bca/3598919)
Supplement: Supplementary file 1 — Supporting Information Additional supporting information can be found online in the Supporting Information section. [file BCA-2026-3598919-s001.docx]

**Supplementary Information**

Supplementary Table 1: Primer sequences for qRT‑PCR analysis

Supplementary Table 2: Antibodies used for Western blot analysis

Supplementary Figure 1: Quantitative analysis of bioluminescence imaging after sodium selenite treatment of BHP10‑3SCp cells expressing a dual‑reporter gene system

Supplementary Figure 2: Uncropped Western blot images for Figure 2

Supplementary Figure 3: ^131^I clonogenic assay in functional dependency of NIS with scrambled siRNA or NIS siRNA treatment in BHP10‑3SCp cells. (A) Representative images. (B) Quantitative analysis of the 1^31^I clonogenic assay. Survival fraction (%) was expressed as mean ± SD. ***p < 0.001, *p < 0.05 (vs. scrambled siRNA); ### p < 0.001 (scrambled siRNA + 131I vs. NIS siRNA + 131I) (Student’s *t*-test).

Supplementary Figure 4: Uncropped Western blot images for Figure 4

| **Name** | **Forward primer** | **Reverse primer** |
| --- | --- | --- |
| *NIS* | CTGCCCCACTCCAGTACATGCC | TGACGGTGAAGGAACCCTGAAG |
| *PAX-8* | ATCCGGCCTGGGATGATAGG | TGGCGTTGTTAGTCCCCAATC |
| *TTF-1* | CGCGTTTAGACCAAGGAAC | GAGTGTGCCCAGAGTGAAG |
| *TPO* | GGAGTCTCGTTGCTCTAGCGT | CTCTGCACTGTGGCGTACAT |
| *TSHR* | GGAATGGGGTTGTCGTCTCC | GCGTTGAATTACCTTGCAGGT |
| *β-Actin* | GCACAGAGCCTCGCCTT | GTTGTCGACGACGAGCG |

**Supplemental Table 1. Primer sequences for qRT‑PCR analysis**

| **Name** | **Dilution Ratio** | **Manufacturer** |
| --- | --- | --- |
| NIS | 1:4000 | Novus Biologicals |
| PAX-8 | 1:4000 | Santa Cruz |
| TTF-1 | 1:4000 | Santa Cruz |
| TPO | 1:4000 | Santa Cruz |
| TSHR | 1:4000 | Santa Cruz |
| p-ERK | 1:4000 | Cell Signaling |
| Total ERK | 1:4000 | Cell Signaling |
| p-AKT^Ser473^ | 1:4000 | Cell Signaling |
| p-AKT^Thr308^ | 1:4000 | Cell Signaling |
| Total AKT | 1:4000 | Cell Signaling |
| p-GSK-3β^Ser9^ | 1:4000 | Cell Signaling |
| GSK-3β | 1:4000 | Cell Signaling |
| p-β-catenin | 1:4000 | Cell Signaling |
| β-catenin | 1:4000 | Cell Signaling |
| Histone H3 | 1:10000 | Cell Signaling |
| Caveolin-1 | 1:10000 | Cell Signaling |
| GAPDH | 1:10000 | Santa Cruz |
| β-Actin | 1:10000 | Cell Signaling |

**Supplemental Table 2. Antibodies used for Western blot analysis.**


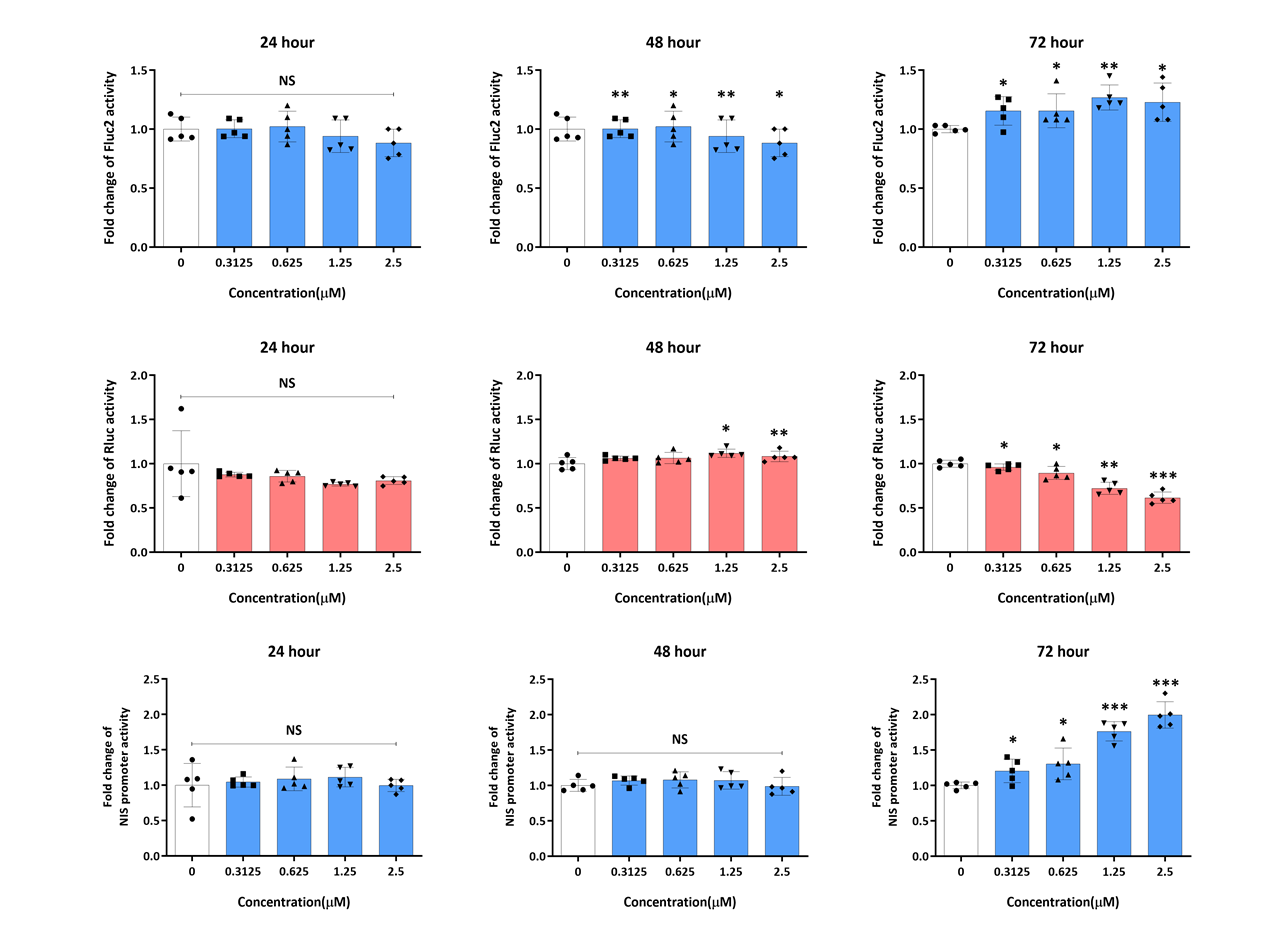


**Supplementary Figure 1. Quantitative analysis of bioluminescence imaging after sodium selenite treatment of BHP10‑3SCp cells expressing a dual‑reporter gene system.**


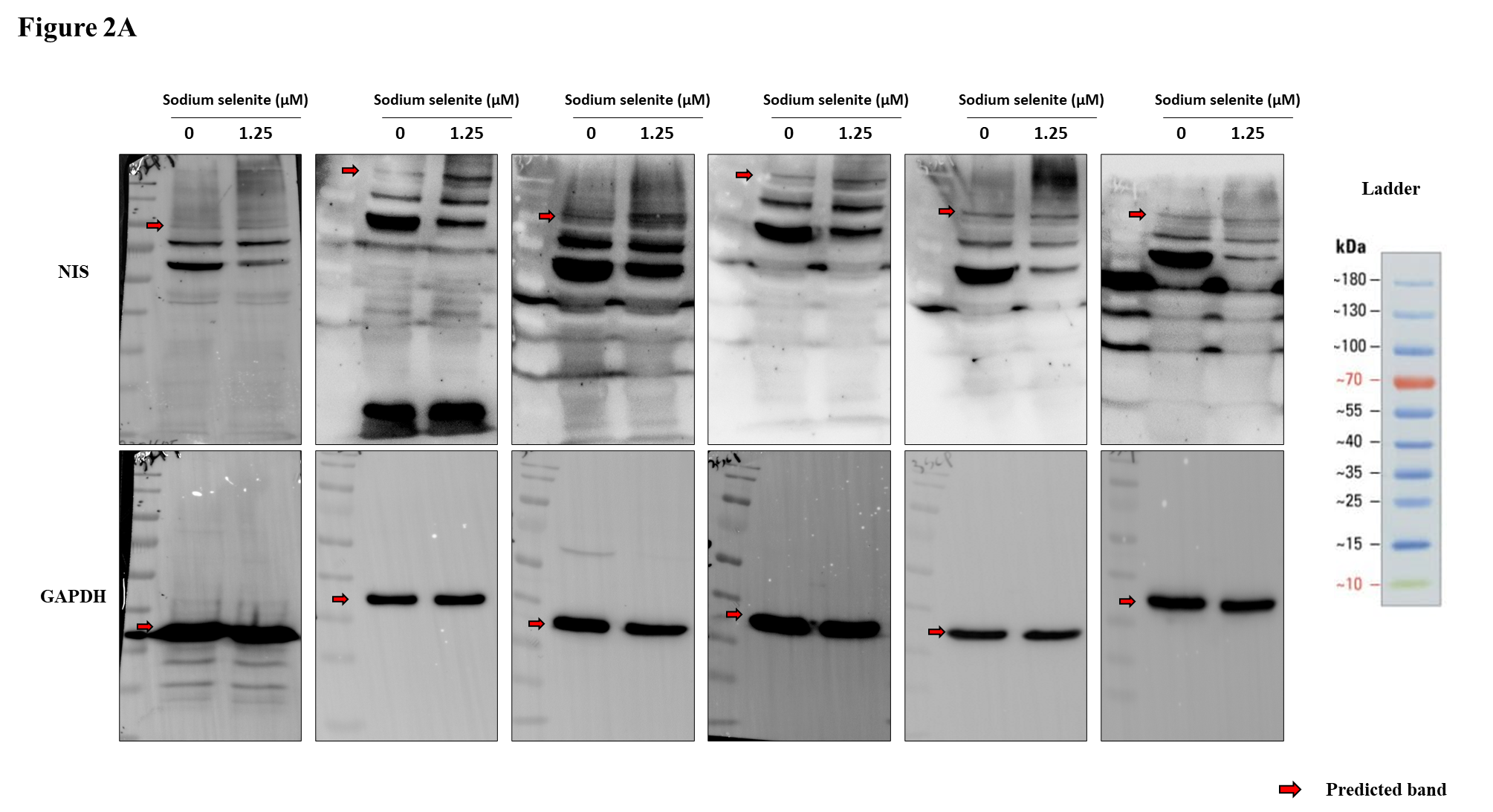


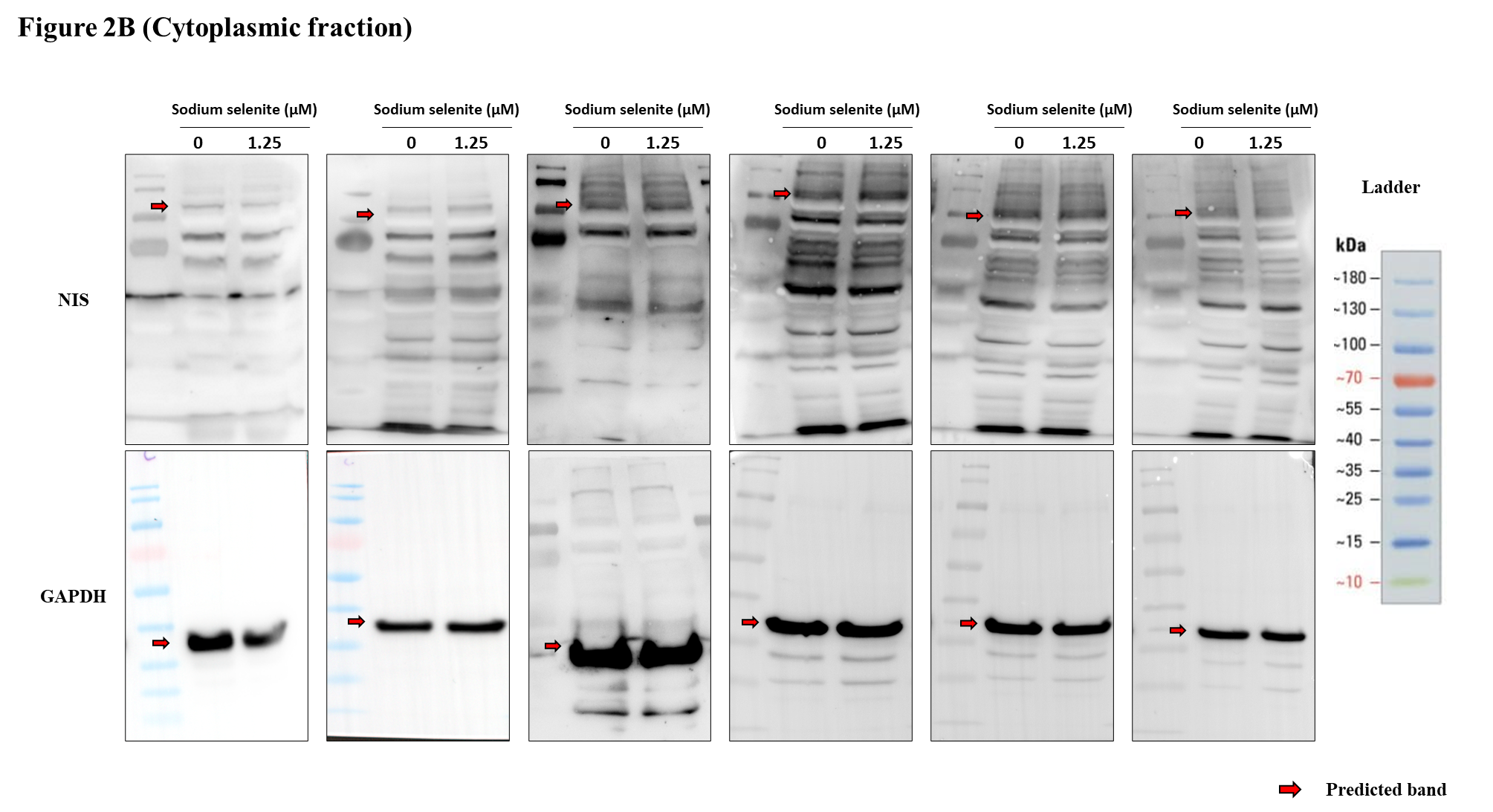


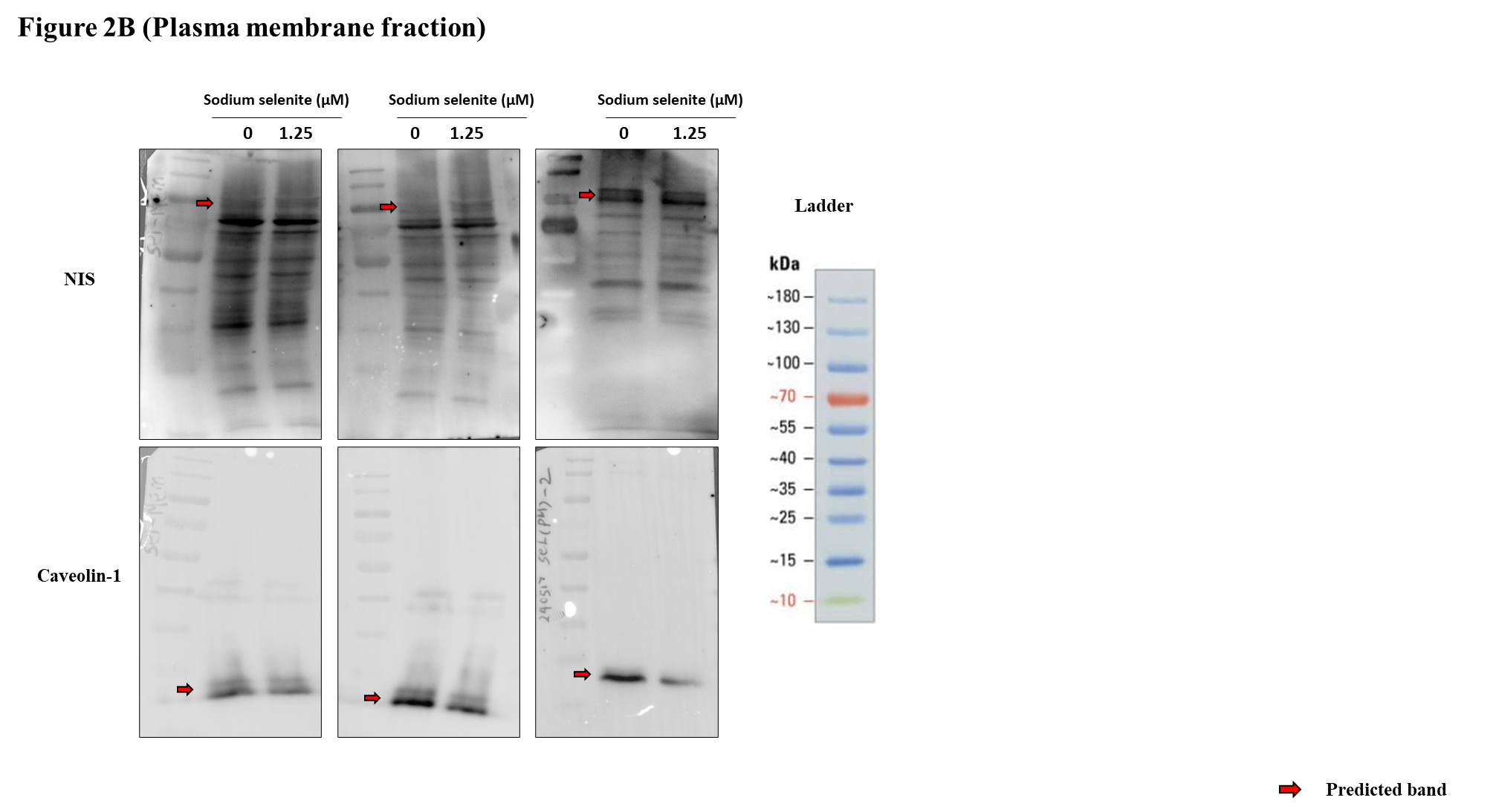


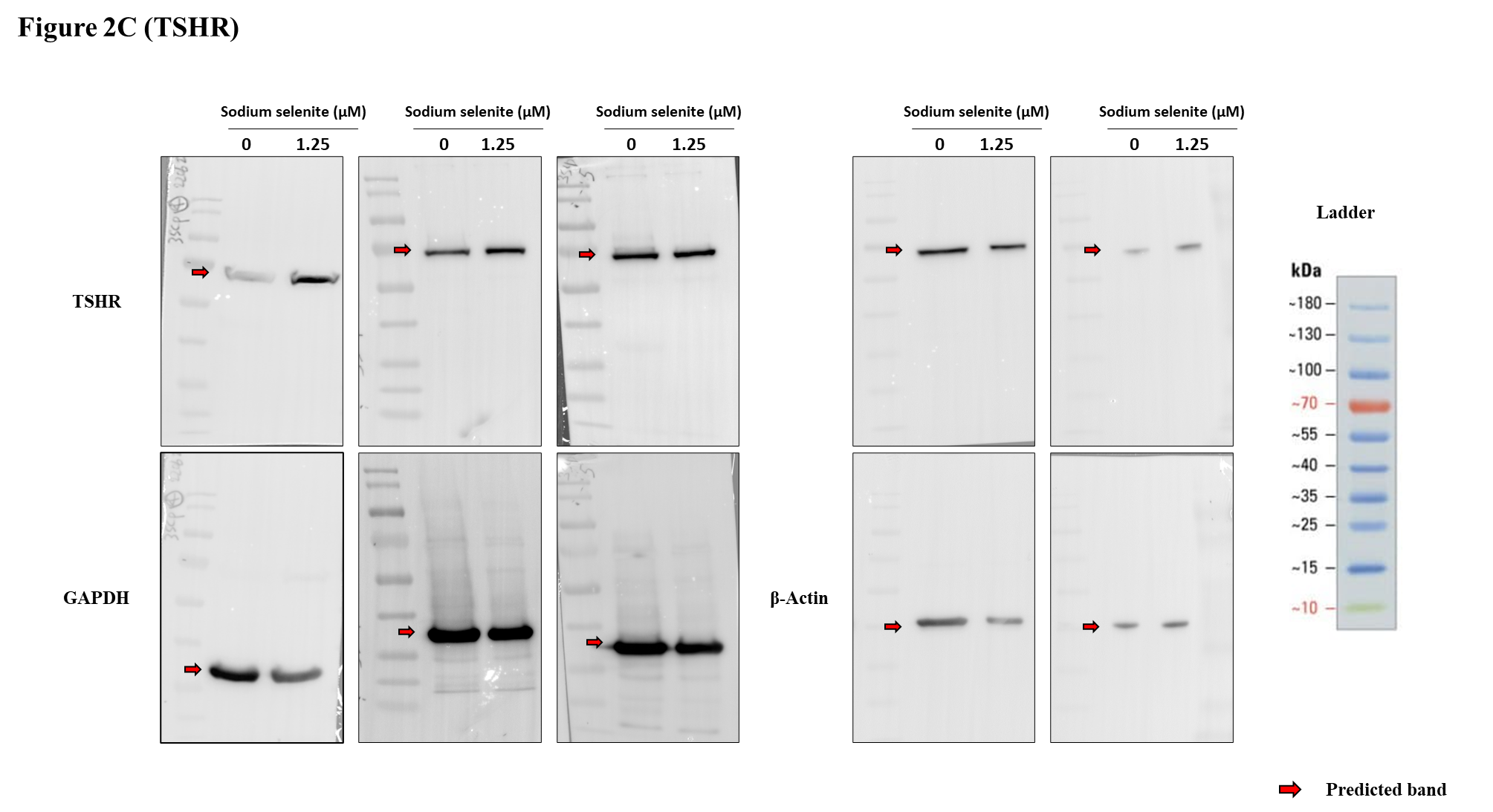


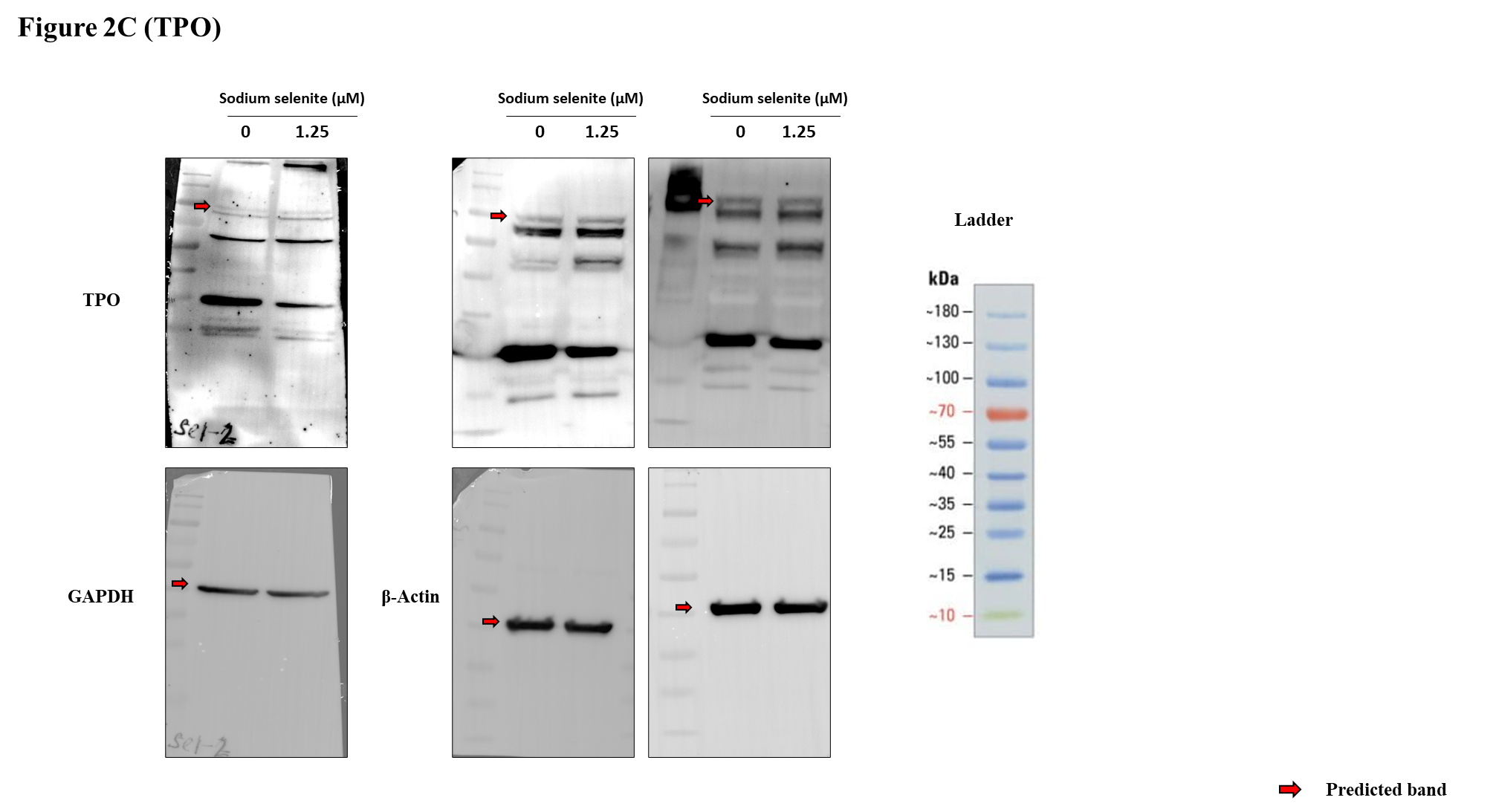


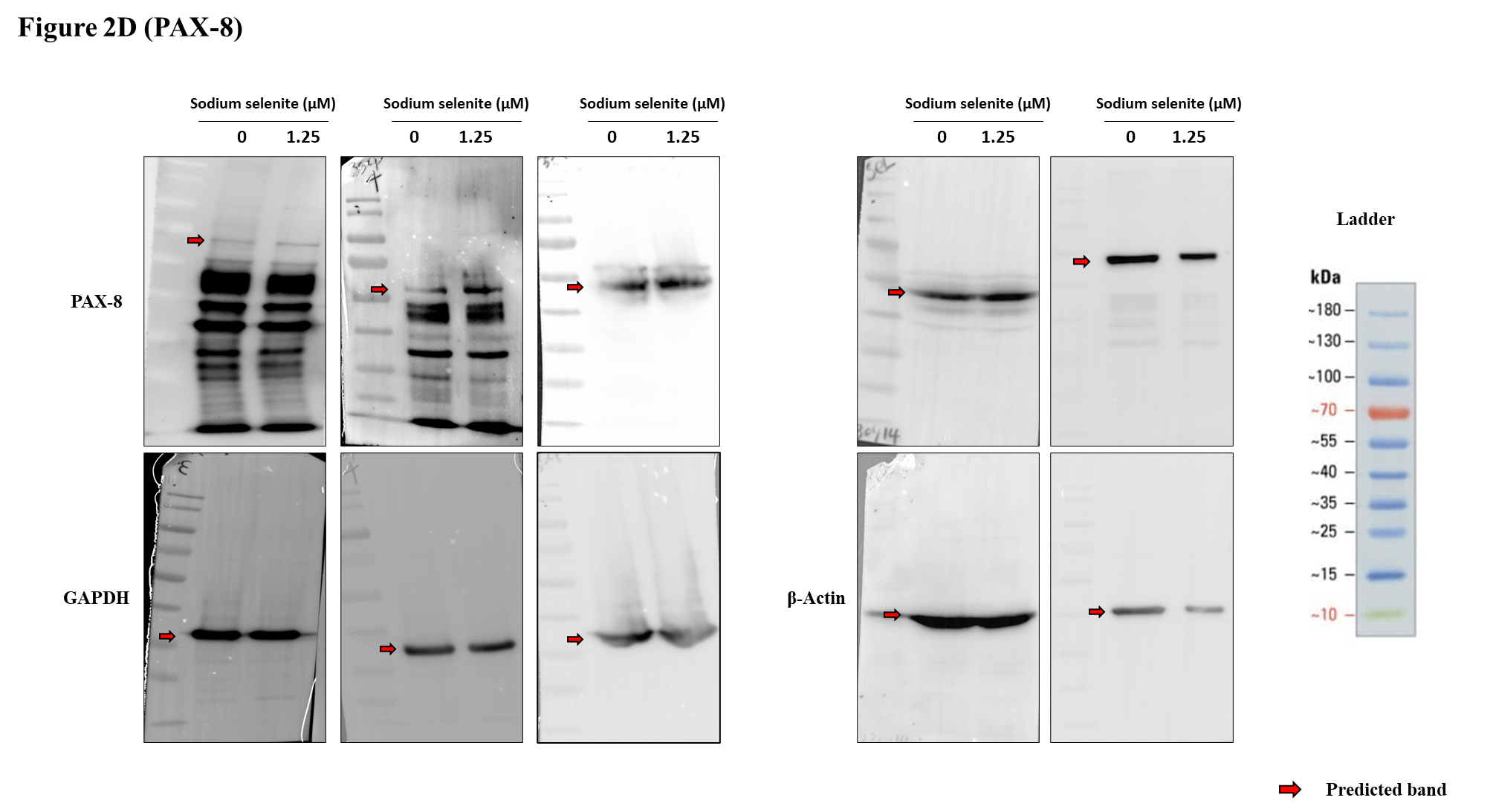


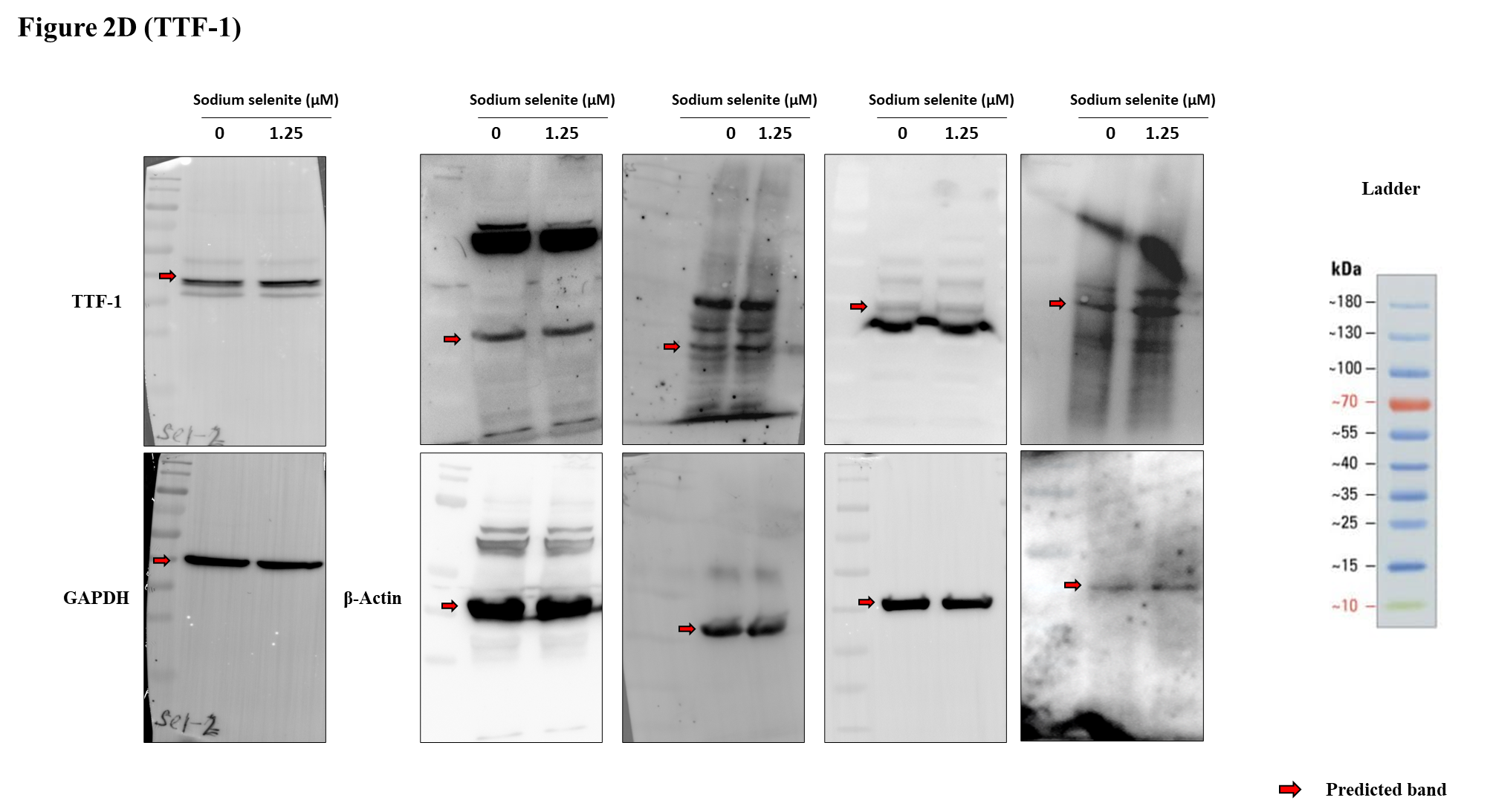


**Supplementary Figure 2. Uncropped Western blot images for Figure 2**


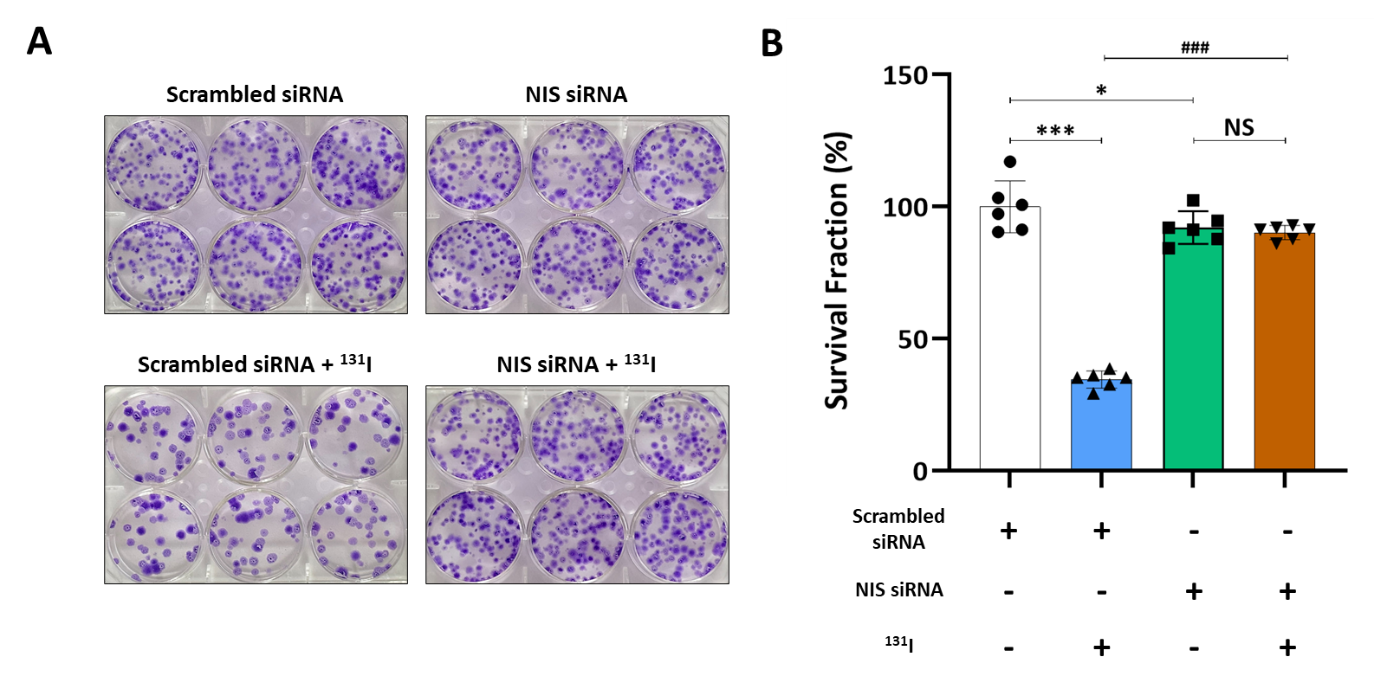


**Supplementary Figure 3. ^131^I clonogenic assay in functional dependency of NIS with scrambled siRNA or NIS siRNA treatment in BHP10‑3SCp cells.** (A) Representative images. (B) Quantitative analysis of the ^131^I clonogenic assay. Survival fraction (%) was expressed as mean ± SD. ****p* < 0.001, **p* < 0.05 (vs. scrambled siRNA); ### p < 0.001 (scrambled siRNA + ^131^I vs. NIS siRNA + ^131^I) (Student’s *t*-test).


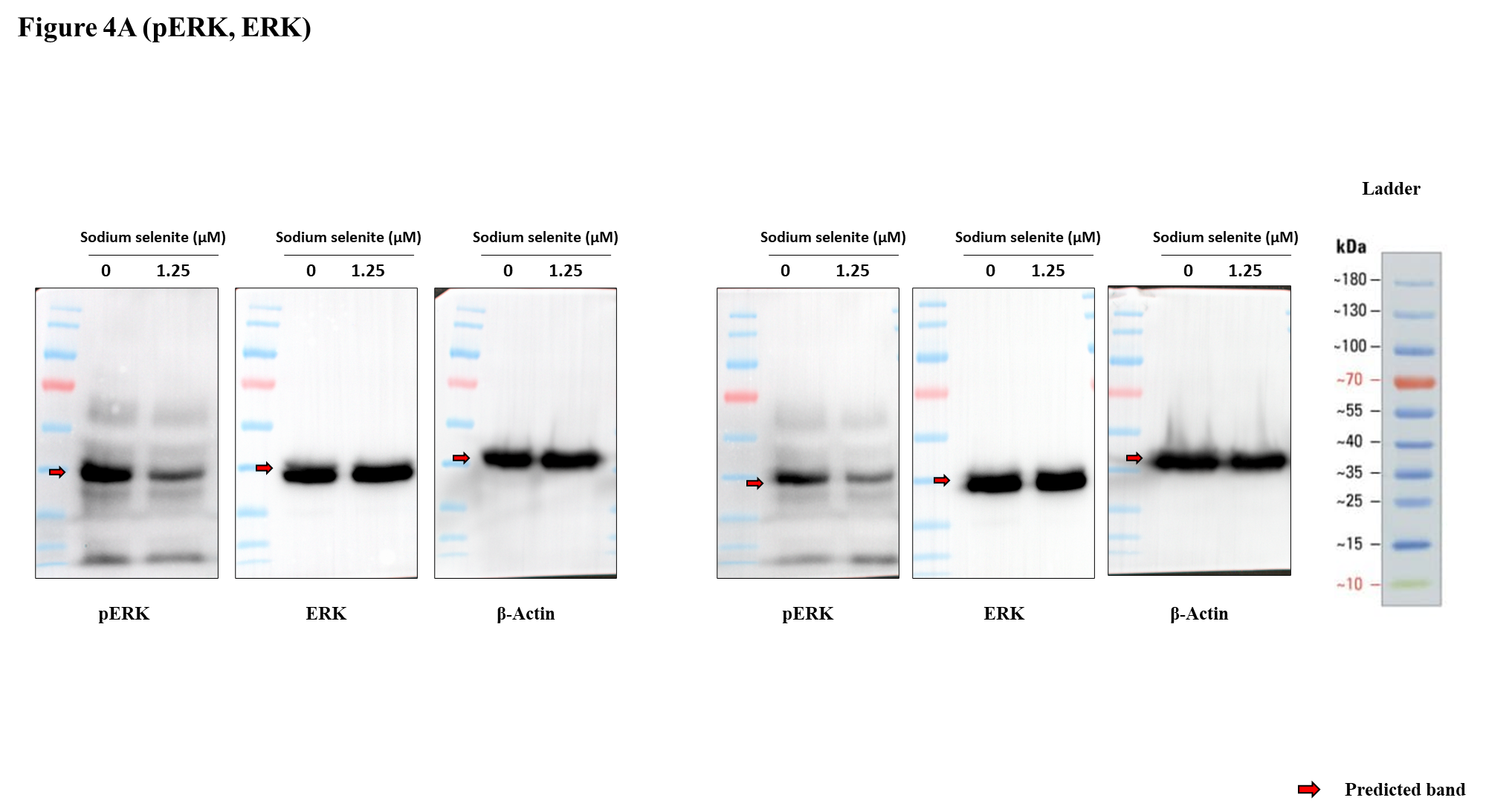


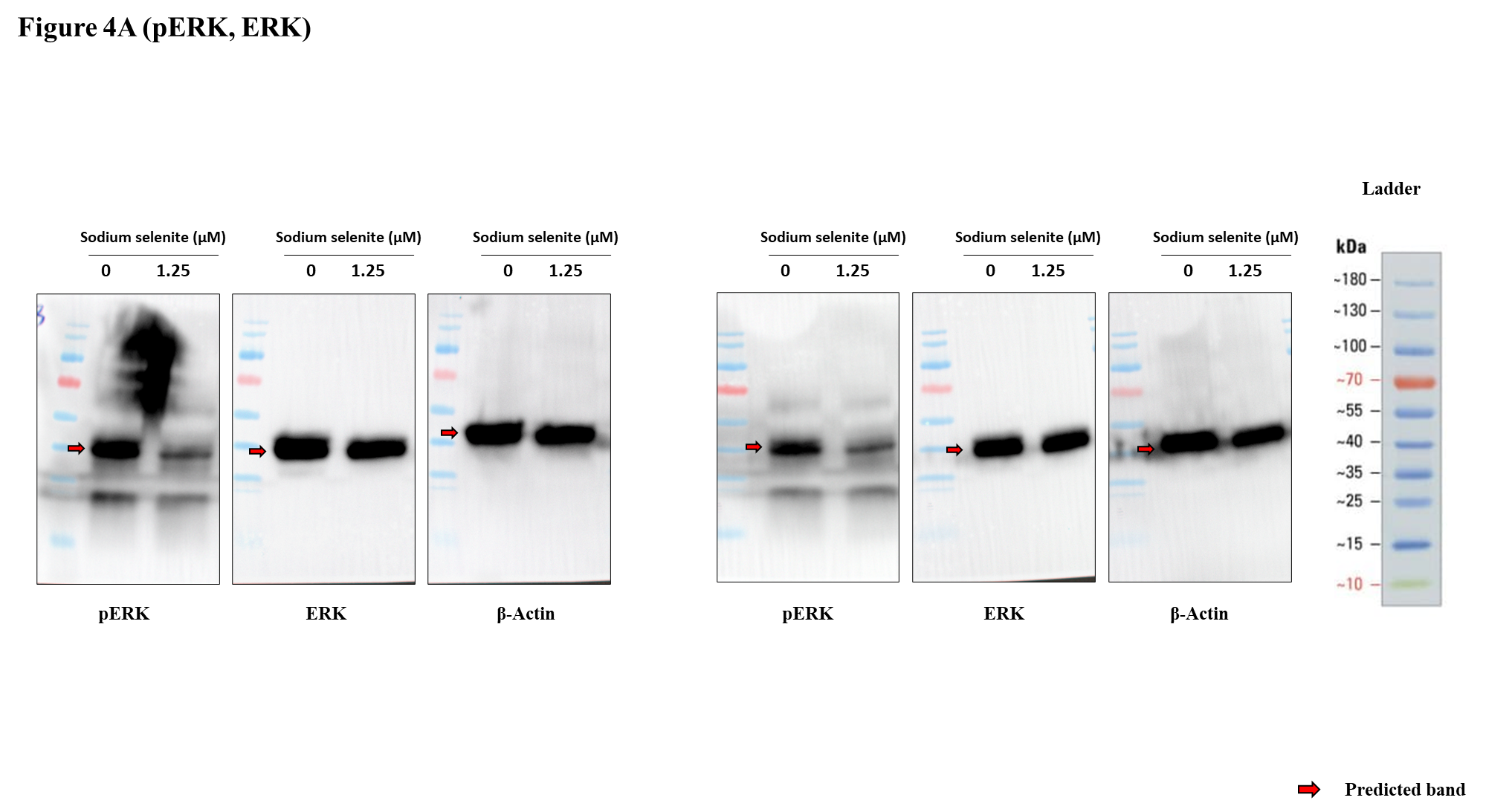


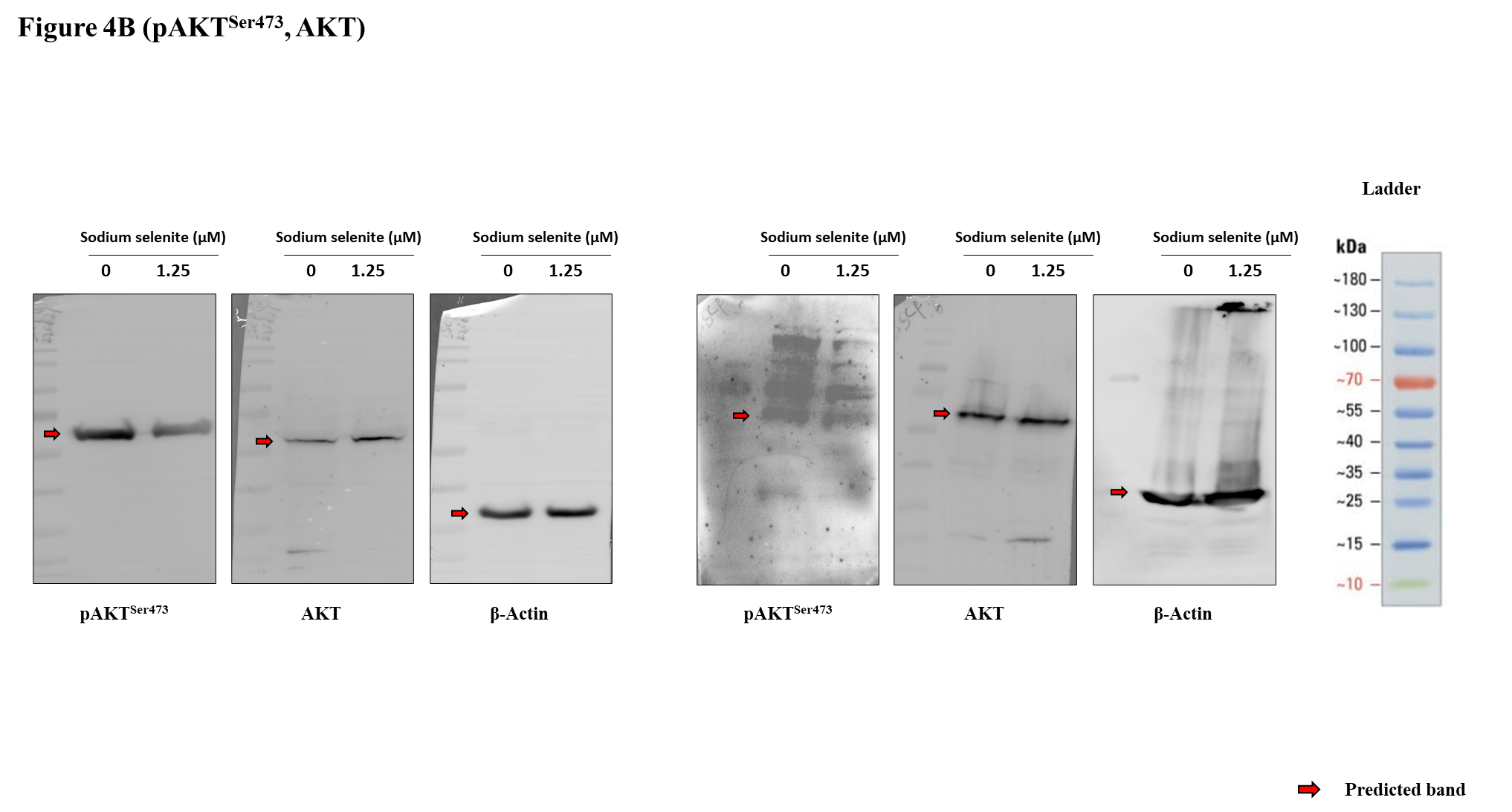


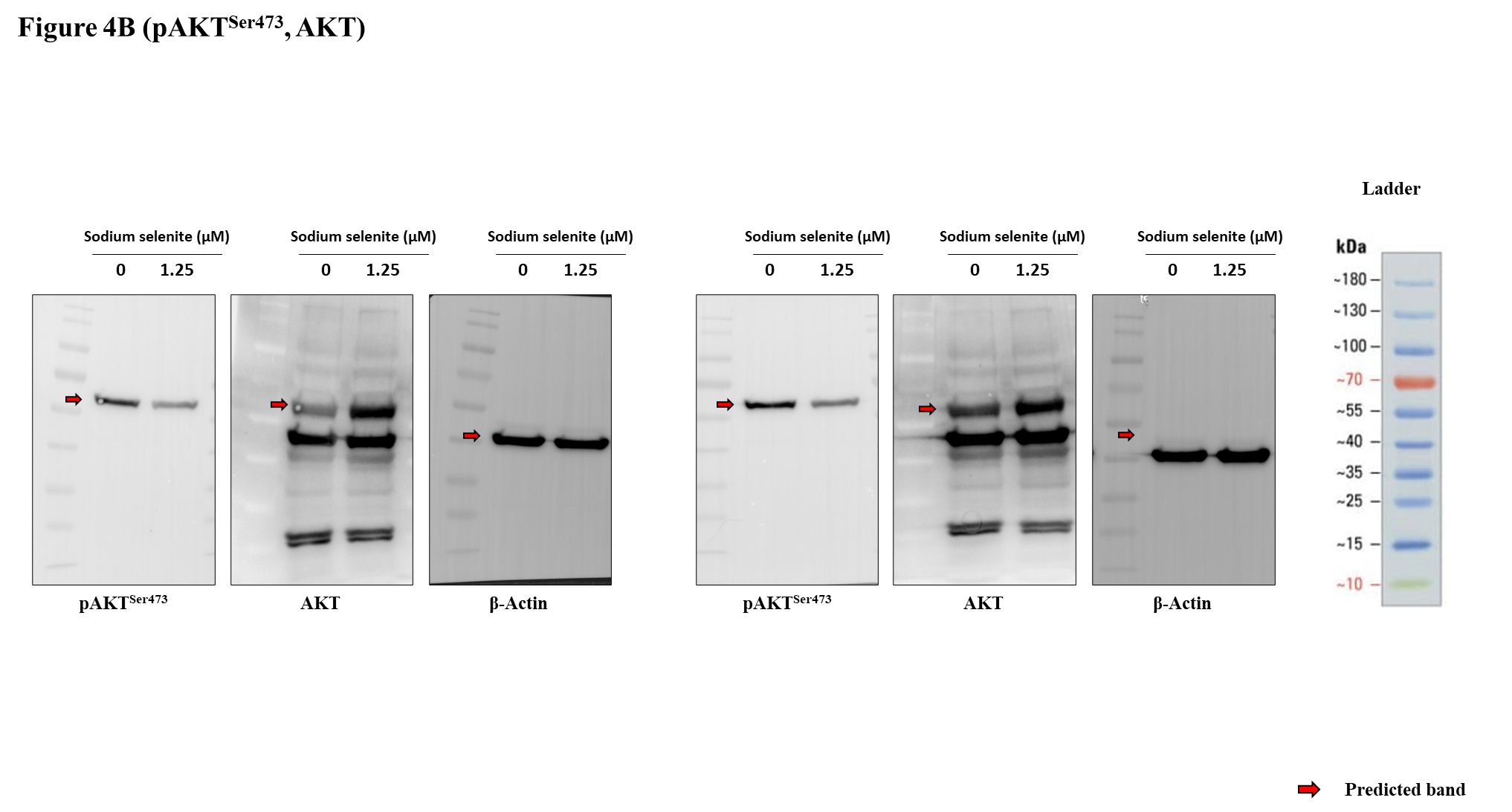


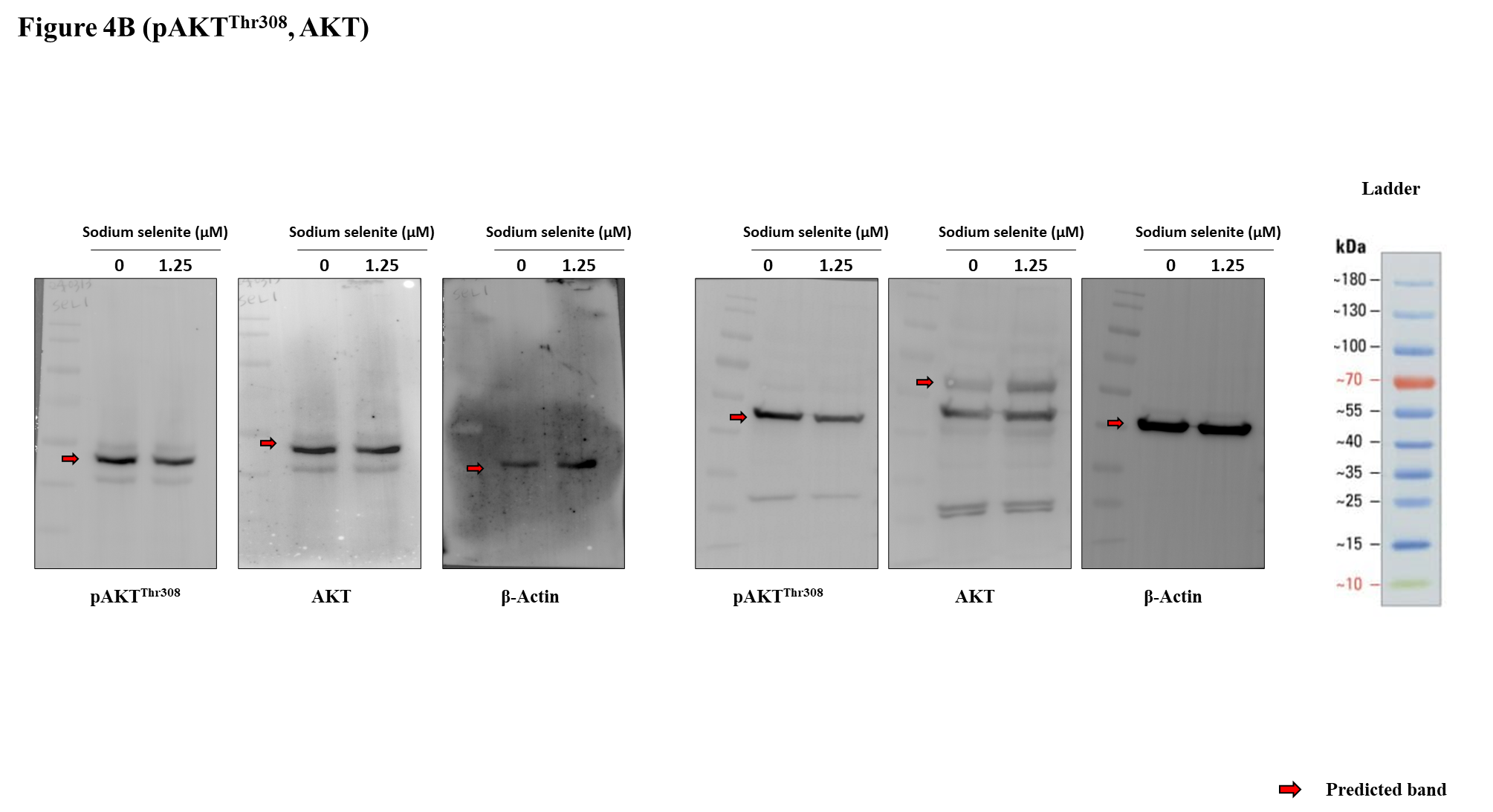


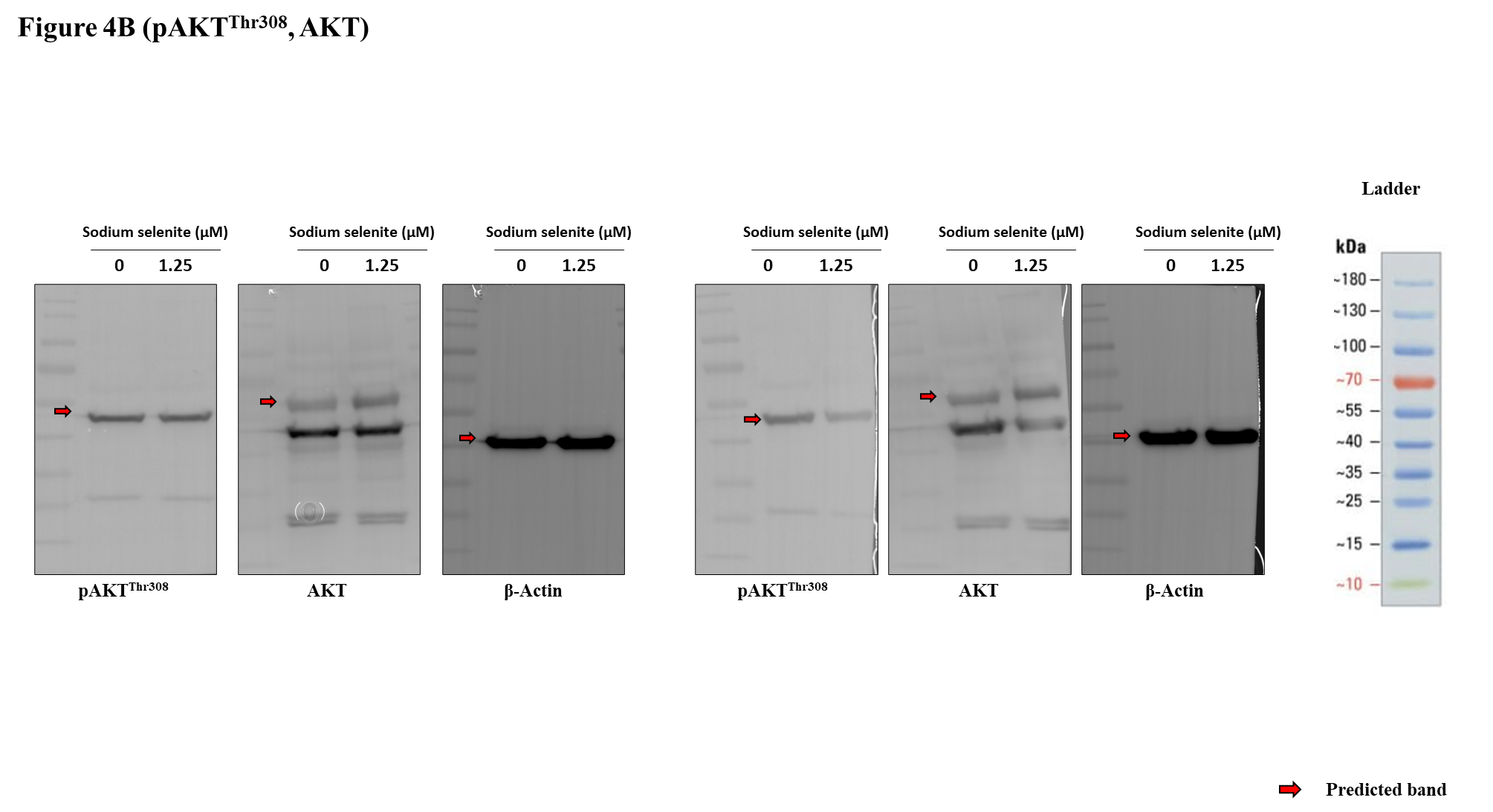


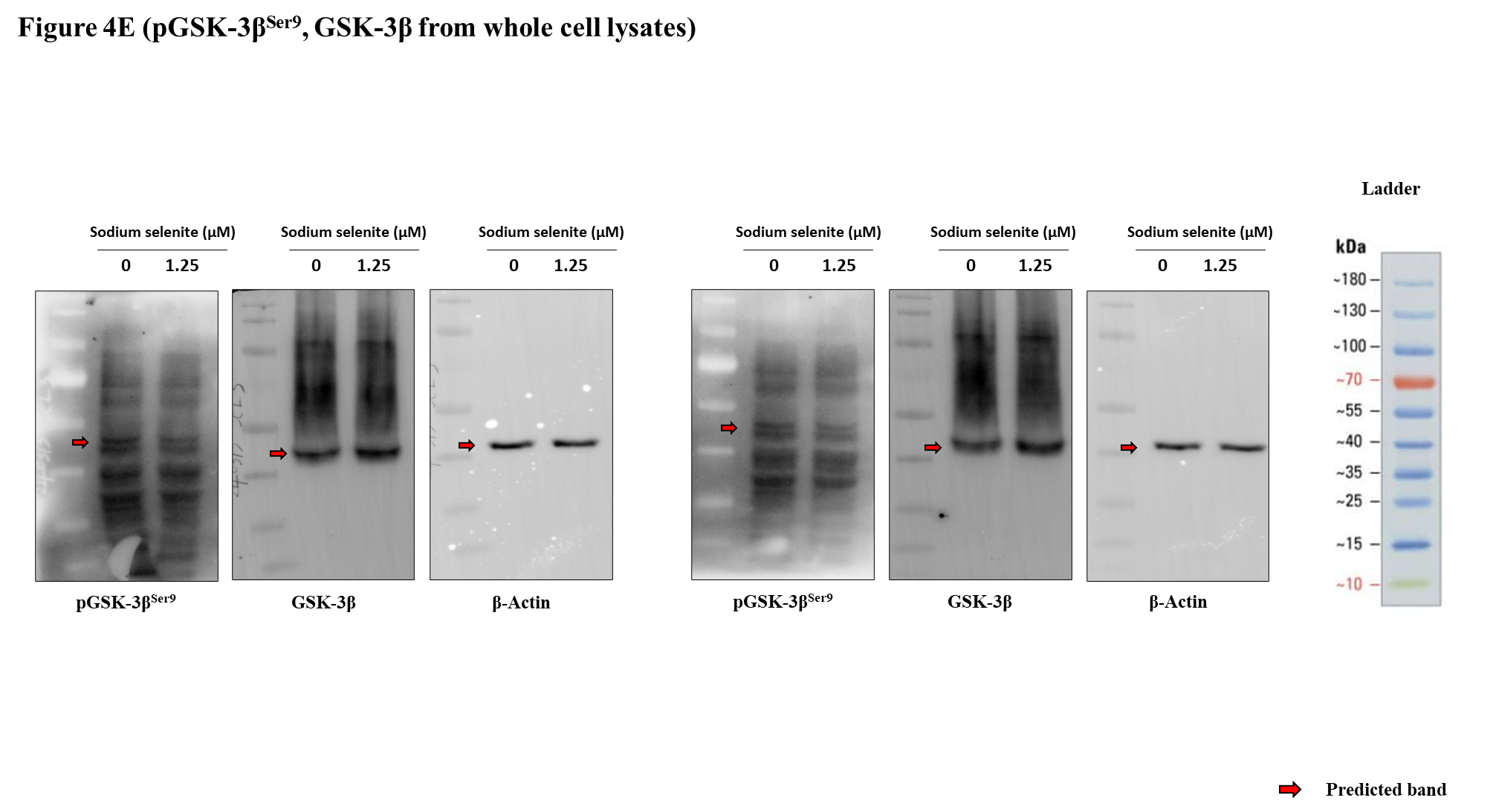


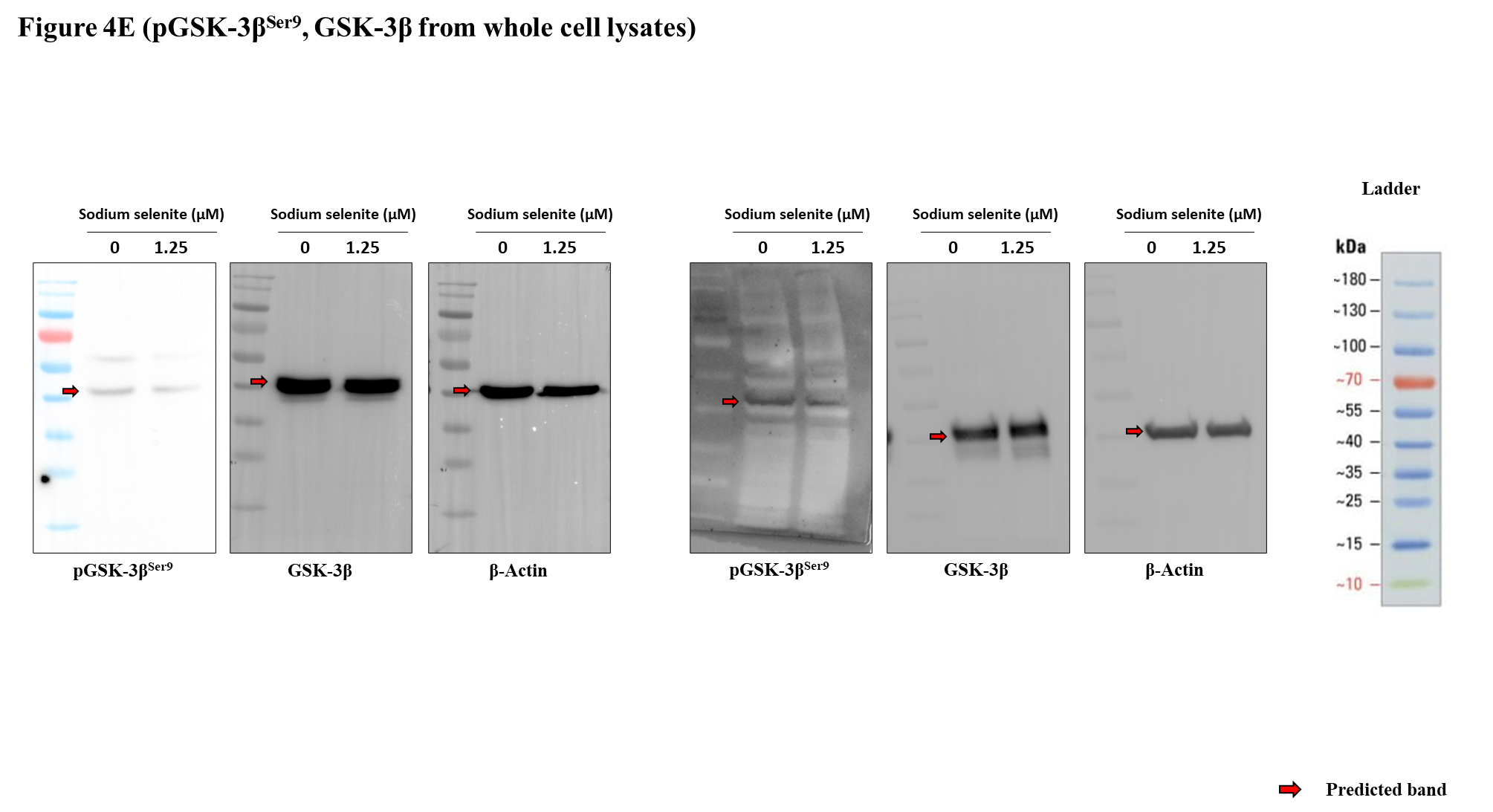


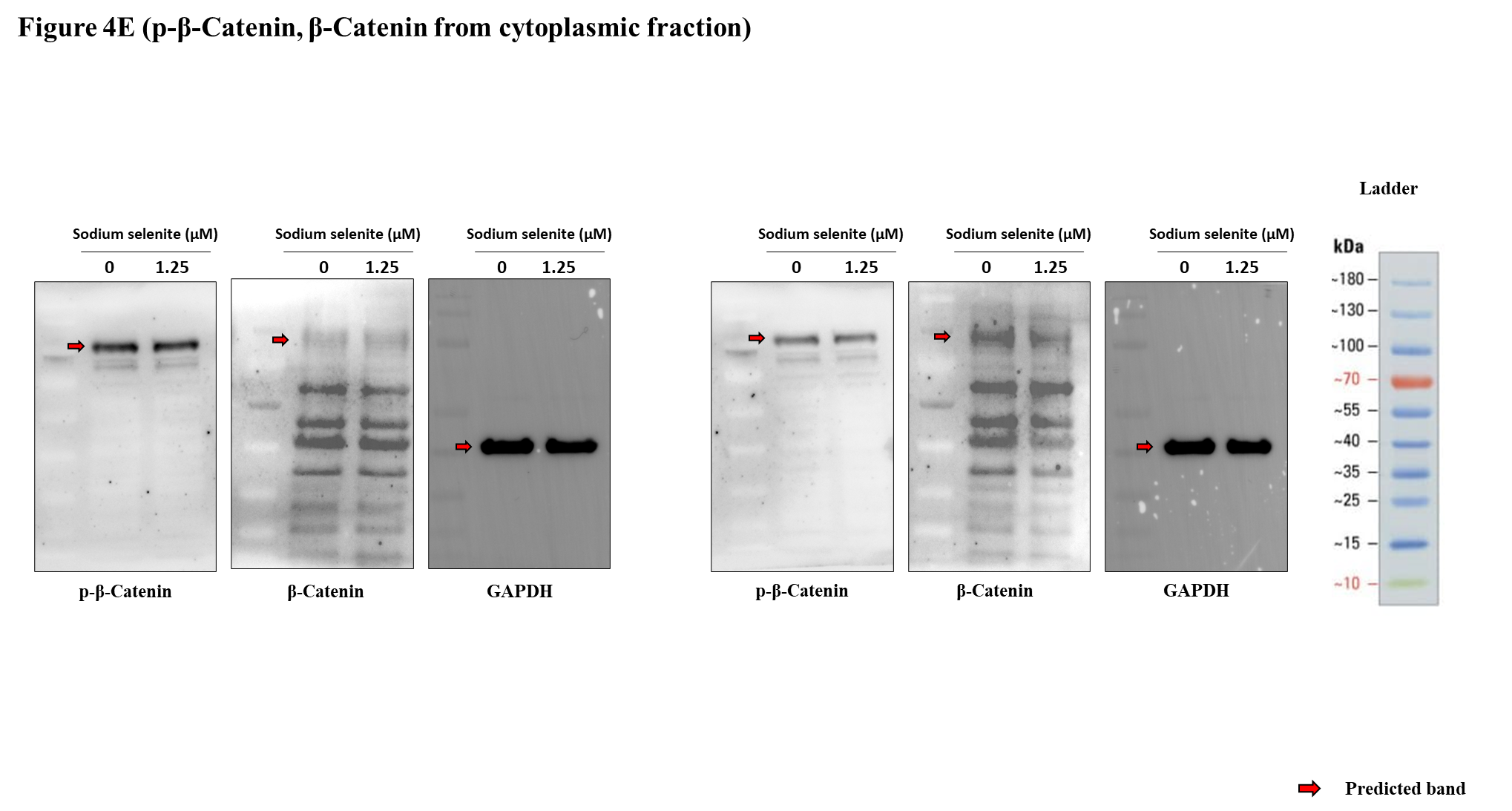


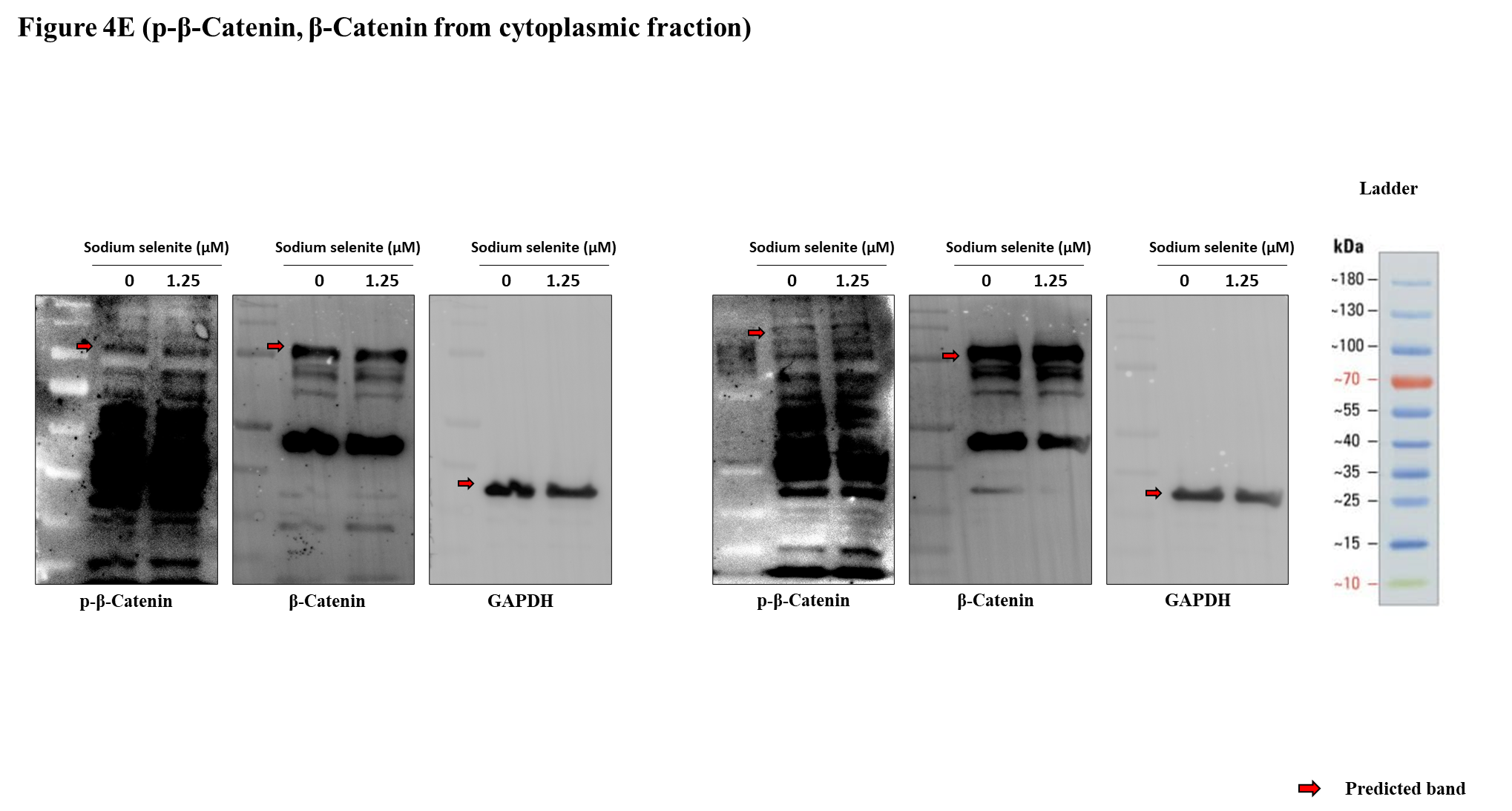


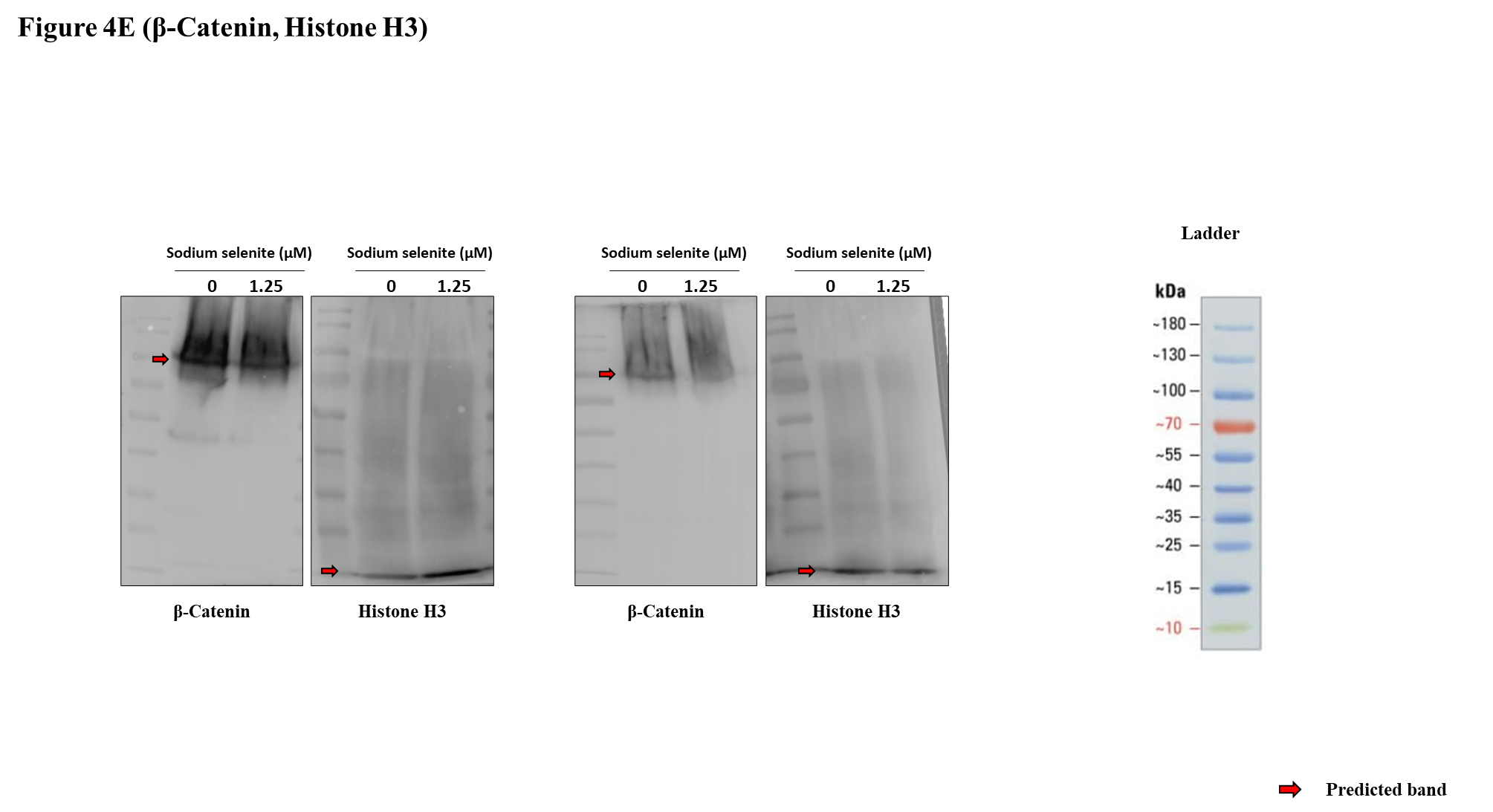


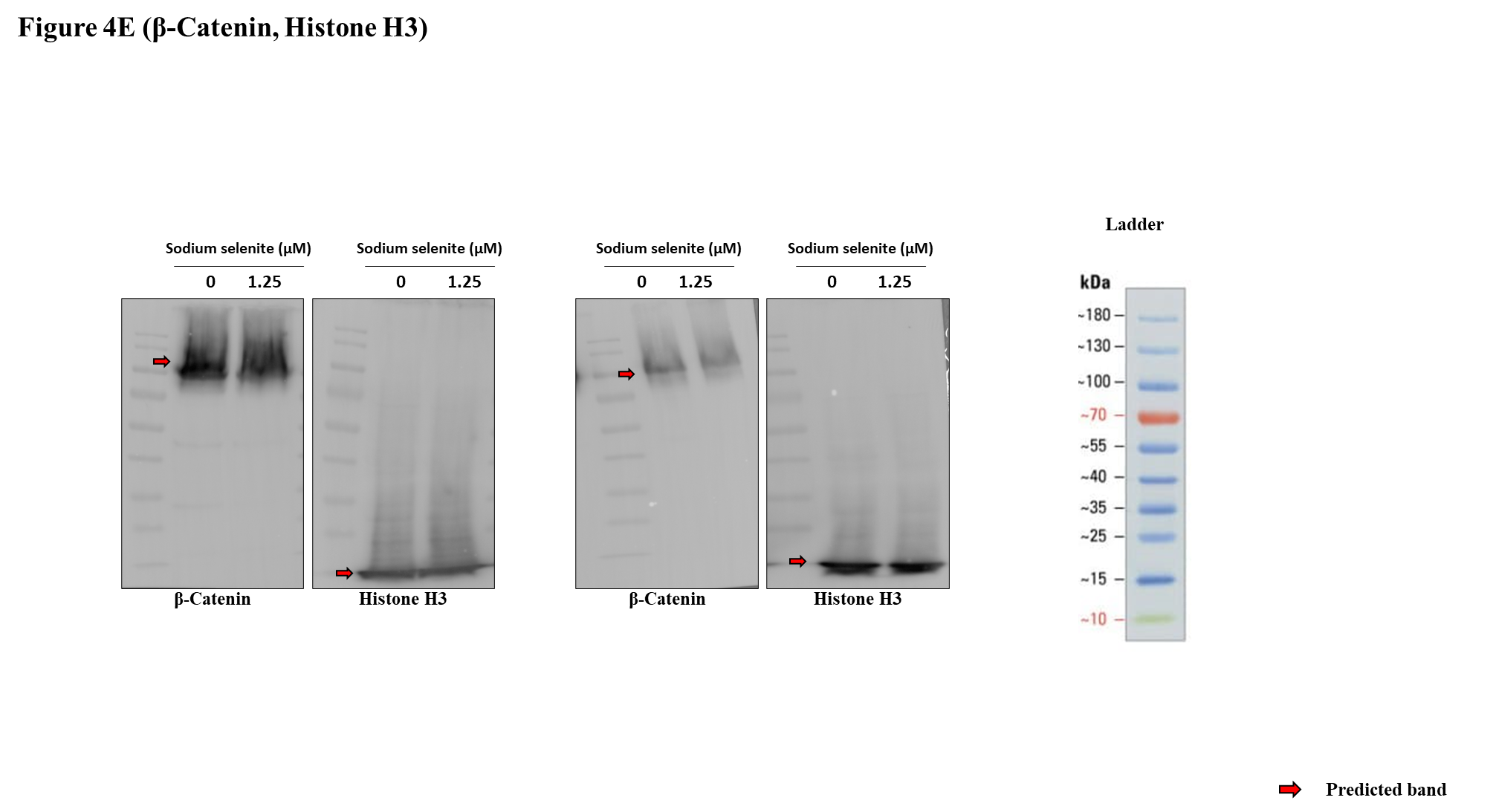


**Supplementary Figure 4. Uncropped Western blot images for Figure 4**
